# Biochar Synthesis from Mineral- and Ash-Rich Waste Biomass, Part 1: Investigation of Thermal Decomposition Mechanism during Slow Pyrolysis

**DOI:** 10.3390/ma15124130

**Published:** 2022-06-10

**Authors:** Rahul Ramesh Nair, Moni Mohan Mondal, Shanmugham Venkatachalam Srinivasan, Dirk Weichgrebe

**Affiliations:** 1 Institute of Sanitary Engineering and Waste Management, Leibniz University of Hannover, 30167 Hanover, Germany; mondal@isah.uni-hannover.de (M.M.M.); weichgrebe@isah.uni-hannover.de (D.W.); 2 Environmental Engineering Department, Central Leather Research Institute, Chennai 600020, India; svsrinivasan@clri.res.in

**Keywords:** biochar, mineral- and ash-rich, waste biomass, pyrolysis mechanism, thermal kinetics, evolved gas analysis, apparent activation energy

## Abstract

Synthesizing biochar from mineral- and ash-rich waste biomass (MWB), a by-product of human activities in urban areas, can result in renewable and versatile multi-functional materials, which can also cater to the need of solid waste management. Hybridizing biochar with minerals, silicates, and metals is widely investigated to improve parent functionalities. MWB intrinsically possesses such foreign materials. The pyrolysis of such MWB is kinetically complex and requires detailed investigation. Using TGA-FTIR, this study investigates and compares the kinetics and decomposition mechanism during pyrolysis of three types of MWB: (i) mineral-rich banana peduncle (BP), (ii) ash-rich sewage sludge (SS), and (iii) mineral and ash-rich anaerobic digestate (AD). The results show that the pyrolysis of BP, SS, and AD is exothermic, catalyzed by its mineral content, with heat of pyrolysis 5480, 4066, and 1286 kJ/kg, respectively. The pyrolysis favors char formation kinetics mainly releasing CO_2_ and H_2_O. The secondary tar reactions initiate from ≈318 °C (BP), 481 °C (SS), and 376 °C (AD). Moreover, negative apparent activation energies are intrinsic to their kinetics after 313 °C (BP), 448 °C (SS), and 339 °C (AD). The results can support in tailoring and controlling sustainable biochar synthesis from slow pyrolysis of MWB.

## 1. Introduction

Biochar is the non-graphitizing porous carbon formed from the progressive dehydration, decarboxylation, dehydrogenation, demethylation, polycondensation, and graphitization (at high temperatures) of biomass in an inert or oxygen-deficient environment. It has versatile applications in carbon sequestration, soil amelioration, energy storage, and gas- or liquid-phase adsorption. The last decade has seen considerable research on the production and applications of biochar derived from wood and other feedstock with low ash and minerals [1,2,3,4,5] and hybridizing them with minerals, silicates, and transition metals to improve their functionalities [6,7,8,9]. However, biomass that is intrinsically rich in these foreign substances is available as a waste by-product of human activities. They are mineral- and ash-rich waste biomass (MWB) from urban areas. As the name implies, MWB differs from conventional lignocellulosic biomass [10] due to their relatively higher concentrations of alkali and alkaline earth metals (AAEM), silicates, and inorganic carbonates that create more ash. It is mainly of technogenic origins, and examples include sewage sludge from wastewater treatment plants, anaerobic digestates from food wastes, and crops grown under chemical fertilization. Biochar derived from such MWB has shown wide applications in adsorption, soil amendment, and carbon sequestration [11]. Synthesizing biochar from them opens new possibilities in the development of renewable multi-functional materials and cost-effective waste management strategies [12].

On the process side, MWB pyrolysis is a complex thermochemical phenomenon with multiple concurrent and/or sequential reactions [13]. Its modeling based on biomass components is not possible due to synergetic effects [14,15] and the catalytic/inhibiting nature of AAEM and inorganics. Kinetic data of biomass in the existing literature cannot be extrapolated for this process design due to (a) erroneous assumptions in modeling and selection of methods associated with solid-state kinetics, (b) considerable physiochemical variations of MWB, even those found in similar regions (e.g., sewage sludge), each with their unique kinetic responses, and (c) lack of consensus in the pyrolysis of even simple biomass components such as cellulose and lignin [16,17,18]. Hence, for the industrial implementation of biochar production, the following subjects must be investigated and correlated with respect to local conditions: (a) the process mechanism, kinetics, and emissions (b) biochar properties (e.g., carbon content, PAH, and heavy metals) according to land application guidelines [19]), and (c) process scaling effects accounting for mass transport phenomena. This study focuses on the subject (a). Common analytical methods for this purpose are TGA-FTIR, TGA-MS, Py-GC-MS, or TGA-MS-FTIR [20]. The physicochemical characterization of the biochar and the influence of process-scaling effects on reaction kinetics are treated in the follow-up part 2 publication.

Herein, the authors investigate and compare the pyrolysis mechanism from three different kinds of MWB: (a) rich in AAEM minerals—K-rich banana peduncle; (b) rich in ash (including heavy metals)—sewage sludge (SS); (c) rich in AAEM and ash—anaerobic digestate (AD). The main purposes of this study are to determine the different pyrolysis stages including secondary tar cracking reactions, their associated gas/volatile emissions and heat flow, kinetic triplets, and reaction thermodynamics using linear and non-linear isoconversional methods. This is to tailor the properties of biochar generated from MWB for its application.

## 2. Materials and Methods

### 2.1. Materials Used

The substrates are collected, oven-dried (at 105 °C), and sampled as detailed in a previous study [21]. The average particle size is kept at 0.2 mm to minimize the effect of transport phenomena [4]. The densities of BP, SS, and AD are 442.92, 964.44, and 624.38 kg/m^3^, respectively. Their high H/C molar ratio (1.51, 1.67 and 1.77 for BP, SS and AD, respectively) and AAEM concentration strengthen the possibility for synergetic interactions during pyrolysis [1]. The results from proximate, elemental, and fiber analysis are shown in Table S1 (Supplementary Material).

### 2.2. TGA-DSC

Pyrolysis characteristics are analyzed using a TGA/DSC 3+ LF thermogravimetric analyzer (Mettler Toledo, Greifensee, Switzerland). Instrument calibration is performed as in [21]. About 10 ± 1 mg of each sample is loaded in a 70 μL alumina crucible without compression. Any type of compression at the time of sample loading can influence their effective bulk density. The runs are carried out at a linear heating rate of 15 °C/min under a nitrogen purge of 70 mL/min (including cell gas flow of 20 mL/min) to the highest treatment temperature (HTT) of 1000 °C. A pre-purge with nitrogen (100 mL/min for 10 min) minimizes the presence of oxygen in the reactor. Duplicate trials are performed for each run and averaged for plotting the thermograms. DSC curves are plotted with blank-corrected (against empty crucible runs) measurements. The heat of pyrolysis (HoP—kJ/kg) at a given temperature is calculated (MATLAB R2020b) as the cumulative mathematical area under the mass-normalized DSC (W/g) vs. time (s) curve.

### 2.3. FT-IR

Online FT-IR measurements for TGA runs are made with the Thermo Fisher Scientific (Waltham, MA, USA) Nicolet 50 spectrometer (DTGS KBr detector) in the range of 4000–400 cm^−1^. Scans are made with a resolution, optical velocity, aperture, and gain of 8, 0.4747, 100, and 1.0, respectively. Each measurement is preceded by background scans. The final spectra are baseline corrected and smoothed in OMNIC 9.7 to generate the chemigrams (profiles of functional groups vs. time) based on the band assignments in Table 1. For each MWB, emissions can be semi-quantitatively determined (as a percentage of total emissions) by calculating the ratio of baseline-corrected area under each chemigram in Table 1 to the total area.

### 2.4. Kinetic Measurements

Nonisothermal isoconversional methods (at six linear heating rates—5, 7, 10, 12, 15, and 20 °C/min) are used for kinetic analysis [18]. Trials are performed as in Section 2.2. Generally, a 1–20 mg sample size is recommended [22]. However, small sample masses may (a) misrepresent heterogeneity in shredded biomass, (b) cause unreliable heat-flux DSC signals, and (c) pose poor correlation with practical pyrolytic reactions due to higher surface to bulk ratio in TG [23]. Meanwhile, large sample masses can increase the thermal lag/inertia between the sample and reference temperature [24] and magnify diffusion effects. Used crucibles are cleaned by successive washing with water and 25 vol % HCl and flaring at 1200 °C. TG data are averaged from duplicate trials. Instead of manufacturer software, MATLAB R2020b is used for data processing and numerical evaluation, as the former lacks clarity on the calculation algorithm and is closed source [25]. The theoretical background of kinetic analysis is explained in Section S2.3 of the Supplementary Materials.

### 2.5. Statistics

The TGA has a temperature accuracy and precision of ±0.3 K and ±0.2 K, respectively. The mass balance has an accuracy and precision of 0.005% and 0.0025%, respectively. Despite being heterogeneous material, the standard deviation for mass data for the substrates is within ±1.5 mg, and that for the heat flow is under ±2.7 mW/mg. (Table S2). The kinetic analysis is performed as per the recommendations and guidelines of the Kinetics Committee of the International Confederation for Thermal Analysis and Calorimetry (ICTAC) [22,26]. The normalized emission measurements for all the non-condensable gases have a maximum standard deviation of ±2%.

## 3. Results and Discussion

### 3.1. Thermal Decomposition Pathway

**Figure 1:**
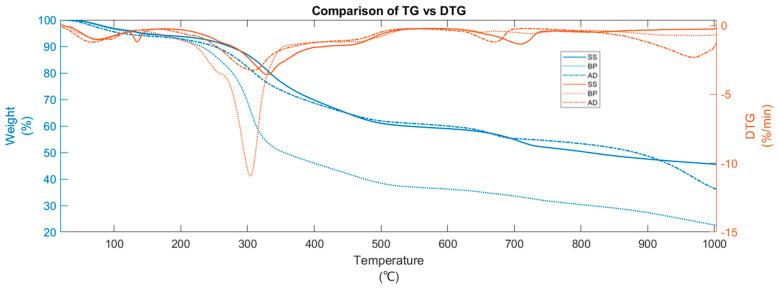
TG–DTG curves during the pyrolysis (at 15 °C/min) of BP, SS, and AD.

**Figure 2:**
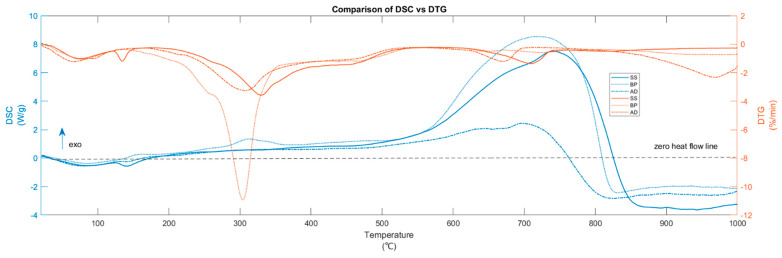
Comparison of DSC and DTG curves during the pyrolysis of BP, SS, and AD.

Figure 1 and Figure 2 show the TG-DTG and DTG-DSC curves during pyrolysis (at 15 °C/min) of BP, SS, and AD, respectively. The cutoff for DTG peak identification [27] is −0.5 wt %/min. The DTG shows prominent peaks below 600 °C for BP and in the entire temperature range for SS and AD. The interpretation of the pyrolytic mechanism begins with a stage-wise investigation of thermal decomposition pathways. After the removal of water through drying, the overlapping process pathways of biomass pyrolysis are broadly classified into multiple stages (Section S2.1).

In BP pyrolysis, the first stage (from 45 to 119 °C) encompasses the evaporation of moisture and the pyrolytic drying [28,29] of water molecules that are physically held by adsorption and adhesion [30]. This is also marked by the endothermic (downward-facing curve) heat flow in the DSC. Active pyrolysis initiates at 156 °C and undergoes a mass loss of 53.68 wt % until the burnout at 519 °C. Here, the shoulder peak at 256 °C and maximum peak temperature (MPT) of 303 °C is due to hemicellulose and cellulose degradation, respectively. Although active pyrolysis usually starts around 200 °C [16], reactions between 150 and 200 °C can include the hydrolysis of extractives [16] and softening of hemicellulose [31]. There is a small exotherm at 150 °C. During active pyrolysis, cellulose devolatilization is endothermic or exothermic (depending on its kinetic route [32,33]), while that of hemicellulose and lignin is exothermic [34]. Pure hemicellulose pyrolysis results in two peaks at around 245 and 295 °C [35]. The first peak arises from the breaking of the branched structures and glycosidic bonds, while the second is from the fragmentation of the ring units [36]. These peaks shift to lower temperatures in the presence of AAEM and merge with those from cellulose. The literature [35,37] shows that from 350 °C to burnout, the broad shoulder peak in DTG arises from the lignin’s ([C_10_H_12_O_3_]_n_) overlapping steady decomposition. This is further confirmed from the exothermic DSC and the featureless D-DSC (seen later in Section 3.3). Lignin can exist as H type (p-hydroxyphenyl C_9_H_12_O_2_), G type (guaiacyl C_10_H_12_O_3_), and/or S type (syringyl C_11_H_14_O_4_) [38]. Depending on its form, lignin can have a wide degradation range from 200 to 800 °C and lacks precise burnout points. Net exothermic DSC during active pyrolysis signifies the influence of hemicellulose and lignin transformation [39] and the favorability of the kinetic route for exothermal char formation of cellulose [38]. The less prominent DTG regions between 650 and 750 °C are from the endothermic decomposition of inorganic carbonates [40,41]. The fact that BP contains them in the least amount makes its influence inconsequential to the total heat generated during the pyrolysis. The increase in heat release after burnout that culminates in the exothermic DSC peak at 725 °C is due to the increase in char aromatization, polycondensation, and dehydrogenation with increasing HTT. The DTG region after 870 °C is small compared to active pyrolysis. Note that even though BP consists of holocellulose and lignin, only a merged DTG peak is seen during active pyrolysis due to the expected synergistic effects in non-synthetic biomass [42].

Compared to BP, the DTG and DSC of SS show visible differences (Figure 1 and Figure 2). This is because of the anaerobic stabilization of the SS, which reduces the aliphatic carbon. After the initial drying from 49 to 119 °C, one more drying stage exists between 122 and 145 °C with a 1.21 wt % mass loss. This is due to the larger presence of chemically bound water that has higher binding energy [30,43]. It is marked by a sharp DTG peak and an associated endotherm at 135 °C. With more chemically bound water, other biomass feedstock [44,45] has shown an extended pyrolytic drying stage up to 150 °C. The onset and MPT of active pyrolysis shifted to higher temperatures—221 °C and 329 °C, respectively, while the hemicellulose shoulder peak fades. The burnout is at 510 °C. This shift toward higher decomposition temperatures is due to the absence of K-induced catalysis, which is low in SS compared to BP. The heat release during the active pyrolysis region (mass loss = 32.57 wt %) is exothermic [46]. However, due to the high ash/VM ratio of 2.47, the exothermicity is lower compared to BP and other SS in the literature [47]. The carbonate decomposition region is more pronounced here relative to BP with MPT of 708 °C and mass loss of 5.76 wt %. Among the three substrates, SS has the largest mass loss in this region since it contains the most calcium carbonates (Table S2).

For AD, the moisture evaporation and pyrolytic drying (5.06 wt % mass loss) are between 37 and 120 °C—similar to BP. The onset, MPT, and endset of active pyrolysis (mass loss = 31.03 wt %) are at 196, 307, and 499 °C, respectively. Like SS, the hemicellulose shoulder peak is not prominent. However, the broad shoulder peak of lignin is well defined. With a total mass loss of 31.03 wt %, the heat release remains exothermic. AD also has about 19.1 wt % of volatiles that are not lignocellulosic—recalcitrant proteins and fatty acids—which are known to cause endothermic decomposition [48] between 200 to 250 °C. These can also be seen as multiple endothermic peaks in the D-DSC curve (discussed later). Thus, although AD and SS are both anaerobically stabilized, AD is less exothermic between 200 and 250 °C. In addition, SS and AD have lower exothermicity during active pyrolysis compared to BP. In fact, this can be attributed to (a) lower cellulose content; (b) the endothermic decomposition of conditioning agents salts such as ferric chloride and lime added to SS and AD before digestion [49] whose presence is also seen from the 7.6 wt % of Cl in AD, 7.4 wt % and 5.39 wt % of Ca in AD and SS, respectively; and (c) less polycondensation and HeSTR compared to BP. Such a smaller heat release of feedstock rich in silicates and minerals is also seen in other studies [46,50]. The DSC after active pyrolysis follows the order BP > SS > AD. The carbonate decomposition region of AD has lower onset (629 °C), MPT (670 °C) and endset (690 °C) compared to SS and undergoes a mass loss of 3.69 wt %, since AD has fewer transition metals to influence decomposition. Thus, a discussion about catalytic effect is warranted for interpreting certain aspects of mass loss.

#### Catalytic Effect of Inorganics

AAEM such as K, Na, Ca, and Mg are inherent in MWB and alter their pyrolysis. Potassium salts are known catalysts during devolatilization and promote exothermic char formation [51,52]. It increases emissions of CO_2_, H_2_O, and CO (by favoring cracking reactions of tar [3]) and NH_3_ (NO_x_ precursor) [53]. The growth of banana plants in Indian soils is heavily dependent on K fertilizers [54]. Consequently, BP has the highest amount of K followed by AD and SS. AAEM in biomass exists in organic (oxalates) and inorganic phases. The latter can have authigenic and/or technogenic origins, which vary considerably amongst different biomasses [55]. Inorganic calcium and magnesium occur in carbonate forms. In raw biomass, potassium, a highly mobile macronutrient, mostly exists in water-soluble and ion exchangeable form—about 80–90%, and the remaining is acid-soluble and residual/insoluble. The predominant water-soluble K may be bound organically (oxalates)—<10% [56] and the rest as inorganics (such as K_2_CO_3_). Biomass drying can also result in the precipitation of about 90% of K as salts (e.g., KCl, KNO_3_, or K_2_CO_3_) [56,57]. KCl and K_2_CO_3_ evaporate and decompose in the ranges of 700–850 °C and 830–1000 °C, respectively [58], while KNO_3_ dissociates around 400 °C [56,59]. Organic bound K (e.g., R-COO-K^+^) decomposes between 200 and 500 °C in the form of K^+^(g). With low or high K:Cl ratios, it can form KCl or KOH (g), respectively.

BP and AD have more than 4–6 wt % K. The catalytic effect of K can also lead to low MPT of holocellulose during the active pyrolysis of BP and contributes to the exothermicity in DSC (Figure 2), which is in agreement with the observations in [16]. After 700 °C, mass loss due to K is significant. However, in BP, there is only a less noticeable release of K. This is because:

1. Most K may be intercalated with carbon in the biochar matrix due to its high electro positivity and the comparative lack of competing electropositive AAEM species such as Na^+^, Mg^2+,^ and Ca^2+^ [60]. This is also an indirect indication of the extent of graphitization of carbon in BP [61]. Furthermore, such intercalated K would be slowly released only at >700 °C [62].
2. At temperatures above 700 °C, some K can be bound to silicates [58], which prevents its release into the gas phase [63]. This is rarely the case with BP as it contains only ≈0.5% silicates.
3. K also can form loosely and tightly bound metal–oxygen complexes [61]. However, the former is less likely at higher pyrolysis temperatures. The latter is less probable compared to AD due to the lower O/C ratio, which is reduced at higher HTT.

In AD, there is a mass loss of 16.30 wt % after 800 °C. Less K is intercalated into the char matrix due to competing electropositive species (Na, Mg, and Ca) totaling about 12.14 wt %, and the presence of chlorine, which provides alternate kinetically favorable reaction routes. In chlorine-rich AD, K is typically present as KCl in biomass [58,64,65].

After 830 °C, K_2_CO_3_ (Equations (1)–(4)) [66] and Na_2_CO_3_ [67] dissociate into alkali oxides and CO_2_ (Equations (9) and (10)). Sodium bicarbonate is a common pH buffer during anaerobic digestion. It is converted to sodium carbonate at lower temperatures (80 to 100 °C) during oven drying. Finally, all alkali oxides (except for LiO_2_, if present) devolatilizes to the respective elements and oxygen [68]. In equations, −ΔH is taken as endothermic.

(1) $$K_{2}CO_{3}⇋K_{2}O\;\left(s\right)+CO_{2}-\Delta H$$

(2) $$K_{2}O\to 2K+\frac{1}{2}O_{2}-\Delta H$$

(3) $$Na_{2}CO_{3}⇋Na_{2}O\;\left(s\right)+CO_{2}-\Delta H$$

(4) $$Na_{2}O\to 2Na+\frac{1}{2}\;O_{2}-\Delta H$$

### 3.2. Evolved Gas Analysis (EGA)

**Figure 3:**
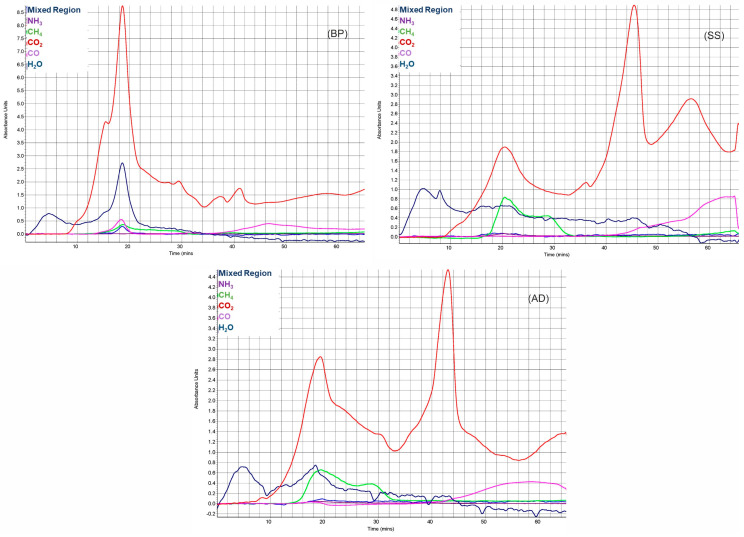
Emission profile of CO_2_, CO, CH_4_, H_2_O, NH_3_ during pyrolysis (at 15 °C/min) of BP, SS, and AD plotted against pyrolysis time.

Owing to the simultaneous evolution of organic compounds with possible secondary cracking, their precise identification is not possible with FT-IR. Hence, they are classified as functional groups of aldehydes and esters [69] called the mixed region. Figure 3 shows the EGA of CO, CO_2_, NH_3_, H_2_O, and CH_4_ during slow pyrolysis of BP, SS, and AD. It is worth noting that the negative absorption of water just indicates the region where these molecules are lower than that in the initial background measurement [70].

In BP, the evaporation and pyrolytic drying are only accompanied by the release of H_2_O (until 120 °C). Then, the CO_2_ evolution begins. The region until 200 °C is from the volatilization of non-polymeric constituents (e.g., sterols), and sugar from biomass. For the pyrolysis of BP, this results in a shoulder peak of CO_2_ at 190 °C [71,72], and it is exothermic (as seen in Section 3.1). In the active pyrolysis zone, the evolution of CO_2_ (from fracture of carboxyl and carbonyl groups), CO (fracture of ether and carbonyl groups), CH_4_, H_2_O (hydroxyl dehydration of holocellulose), and organic functional groups (C-O-C of alcohols and phenols [73] with absorbance between 1200 and 1000 cm^−1^) initiates. They reach a maximum at MPT of 303 °C. The release of organic functional groups from holocellulose is limited to the region under burnout. This further ascertains the inference from Section 3.1 that burnout temperature is the endpoint for holocellulose’s rapid devolatilization. Furthermore, only BP releases more CO during active pyrolysis due to its high cellulose content. The vapor phase reaction of oxygen-containing functional groups also leads to the release of H_2_O here [71]. The CH_4_ is predominantly from the cracking of methoxyl groups of lignin [14,39], which conforms to the DTG data. It continues until the active pyrolysis burnout with the end of the overlapping lignin devolatilization. Until the end of active pyrolysis (519 °C), a trend is seen where the contour variations in the overall absorbance spectra match that of the DTG curve. Then, it alters at higher temperatures where large IR absorbances are seen for <0.5% change in DTG. Between 550 and 685 °C, two CO_2_ peaks are present along with the small evolution of CH_4_ due to the decomposition of oxygen-containing heterocyclics [74] and decarbonylation of phenolics derived from recalcitrant lignin [75]. The CO above 700 °C is from the gasification of char in CO_2_–Boudouard reaction [76]. Similarly, for the three substrates, an increase in CO is always coupled with a decrease in H_2_O. Reasons can be (a) the gasification of char in the presence of water molecules and (b) water–gas shift reaction, which is catalyzed by the presence of AAEM species and by the CO and water molecules present in porous char. However, this correlation of CO and H_2_O needs further investigation, as H_2_, a common by-product here, cannot be detected using FT-IR.

In SS, after initial drying, the extended pyrolytic drying stage is demarked by a second H_2_O absorbance peak at 135 °C—the release of chemically bonded water, as seen in Section 3.1. Then, during active pyrolysis, the release pattern of CO_2_ is similar to BP, while CH_4_ emission from lignin is more pronounced, ending with a shoulder peak at around 520 °C. This finding is consistent with the fact that SS has a higher ratio of lignin to holocellulose. Organic functional group release is lower than BP, as SS has fewer volatiles. The two high-temperature peaks of CO_2_—at 708 and 872 °C—are higher than the ones during active pyrolysis. This illustrates that the predominant generation of CO_2_ for SS is not from depolymerization reactions. Between 644 and 737 °C, CO_2_ contribution is from the decomposition of CaCO_3_, other macromolecular inorganics [77], and aromatic condensation. The endothermic carbonate decomposition is identifiable with CO_2_ arising from its direct decomposition (Equation (5)) as well as CO emission from its decomposition on the char surface (Equation (6)) [41]. Despite this, aromatic condensation causes a net exothermic peak here.

(5) $$CaCO_{3}\to CaO+CO_{2}\;$$

(6) $$2CaCO_{3}+Biochar-C\to 2CaO+2CO+CO_{2}\;$$

In AD, extended pyrolytic drying is absent. At ≈150 °C, like in BP, there is CO_2_ release from sterol and non-polymeric structures. Although there is no corresponding DTG peak, an endotherm is noticeable here. For BP, this was an exotherm. The difference is due to comparatively more water of crystallization release in BP [78,79], which would also explain the higher IR absorbance from H_2_O. Active pyrolysis is characterized by CO_2_, CH_4_ (SS and AD have higher lignin to holocellulose ratio), organic volatiles, CO, and NH_3_. The ether groups of lignin contribute to higher CO emissions [14,80]. The CO_2_ peak between 628 and 690 °C is from carbonates with a corresponding endotherm.

From EGA, the start of exothermic homogenous secondary tar cracking reactions (HoSTR) is indicated by the release of CO after 500 °C during other biomass and sludge pyrolysis [81,82]. Lignin also triggers CO but is coupled with CH_4_ release. For BP, SS, and AD, HoSTR is prominent from ≈565 °C. However, the exact endset of HoSTR can vary between 700 and 1000 °C, depending on reactor and feedstock [51]. Determination is difficult because, at higher temperatures, inorganic decomposition and gasification occur. In addition, this endset depends on the type of tar (secondary or tertiary) formed at these temperatures [83]. However, char formation from HoSTR is less [84] compared to HeSTR [85].

#### 3.2.1. High-Temperature Gasification

Another zone of interest is 700–950 °C, where the in-situ gasification of char in the presence of evolved CO_2_, CO, or H_2_ occurs. A comparison of thermograms and FT-IR chemigrams of open-lid and closed-lid TGA trials can reveal more information about this stage. They are shown in Figures S1–S6 (Supplementary Materials). Above 700 °C, the contribution from tar cracking to evolved gases is only a minor percentage [85]. For SS, these gasifying agents arise from the thermal cracking of some macromolecular compounds [86]. Lignin can also pyrolyze in this region exothermally. However, such a scenario should also be present in closed-lid experiments. When SS was pyrolyzed in crucibles with a closed lid (which increases the residence time of evolved products in contact with biochar-C matrix), CO_2_ and CO peaks disappear (after 775 °C) with a smaller rate of mass loss. This hints at the redeposition of gaseous macromolecules on the char matrix without allowing homogeneous secondary tar cracking reactions to CO and CO_2_. For AD, char gasification reactions in this zone can be explained using the active site theory [87,88,89]. The closed-lid pyrolysis of AD sees higher CO_2_, CH_4_, and CO release between 700 and 950 °C, while that of BP has lower emissions. The gasification reactivity of char depends on process temperature and pressure, porosity, particle size, and active sites. Porosity decreases for non-graphitizing carbon above 700 °C due to carbon stacking and the breaking of interlinks, and it shows less influence on high-temperature chars [90]. In the zone of interest, temperature and pressure may be assumed to be similar for the substrates, since self-heating and variations in ambient pressure during processes are considered negligible. Then, the predominant effects are that of active sites (edge carbon atoms due to the availability of their unpaired σ electron) on the carbon structure and its dependence on defects that create charge imbalances increasing surface reactions, and the AAEM/catalytic dispersion of metal oxide intermediates on the carbon matrix. AD has a relatively high Na and Ca concentration, serving as a catalyst for char gasification with desorbed O from the basal planes. This reaction would be highly exothermic (seen in the DSC of closed-lid pyrolysis of AD) and favors the hydrogasification (also catalyzed by Na) and steam gasification (lower H_2_O release for the AD during closed-lid confirms its higher consumption as a reactant), leading to an increase in CH_4_ and CO_2_. However, K as AAEM does not seem to stimulate catalytic gasification in the pyrolysis of BP because of its char-intercalated form (C_n_K), as seen earlier. The lack of catalytic activity of K, Na, and Ca in SS is due to the inhibiting effect of silicates (SS has [K + Na + Ca]/Si low molar ratio of 0.6) [91]. Furthermore, transition elements, depending on their form, can have a catalytic effect (inferior to AAEM) on gasification as π-electrons from carbon transfer to their d-band, which weakens the C-C bond, as seen in [62].

#### 3.2.2. NO_x_ Emissions

NO_x_ precursors during the slow pyrolysis of biomass, in terms of predominance, follow NH_3_ > HCN > HCNO. During the pyrolysis of these MWB, NH_3_ and HCN emission profiles are very small (Figure 3) compared to permanent gases and volatiles. However, they can pose emission problems during the process scale-up. These precursors depend on the physicochemical characteristic of feedstock and are unaffected by the heating rate [92]. The release mechanism of NH_3_ and HCN, as seen in [93,94], is from the deamination of amide-N (polyamides, proteins) during active pyrolysis—route (a)—and the hydrogenation of heterocyclic-N (embedded in biochar during char formation) and the thermal cracking of amine-N (in tars) during HeSTR—route (b). During slow pyrolysis, route (b) is limited by the feasibility of HeSTR and by the availability of H radicals and volatiles during HeSTR. For SS, AD, and BP, release profiles of NO_x_ precursors are shown in Figure S7 (Supplemental Material). NH_3_ release is mainly detected in the active pyrolysis zone where HCN emissions are comparatively negligible. In this zone, NH_3_ release for SS and AD originates from the rupture of C-NH_2_ bonds in amino acids [95]. This amide-N enters the volatile-N phase and combines with H radicals evolved from the dehydrogenation of aliphatic structures and pyrolytic water [94]. This resulted in less H_2_O release for SS and AD compared to BP. With the highest holocellulose content, BP makes more unstable char-N sites available for HCN release during active pyrolysis [96]. Thus, amongst the three substrates, the HCN peak of BP is highest during active pyrolysis. During the secondary pyrolysis zone, NH_3_ emission is lower but continues for SS and AD through route (b). For BP, NH_3_ release increases from T > 750 °C, which requires further investigation, since the reforming of N in NO (formed from decomposition of potassium nitrates in BP) through hydrogenation is not kinetically favorable.

### 3.3. Heat of Pyrolysis and Biochar Yield

**Figure 4:**
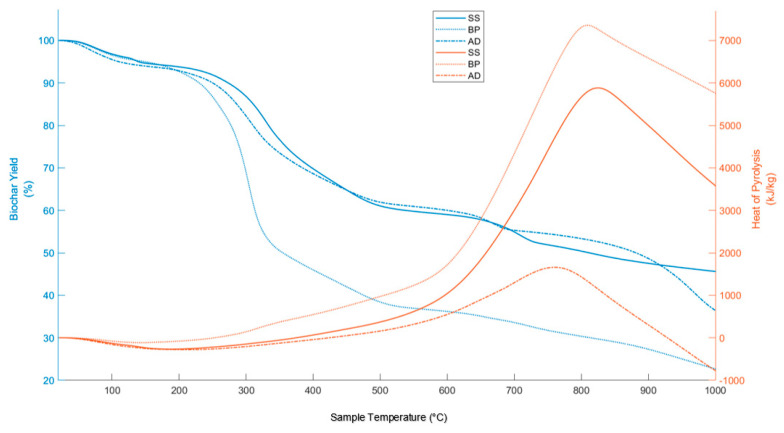
Change in Heat of Pyrolysis (HoP) and biochar yield of BP, SS, and AD with process temperature.

**Figure 5:**
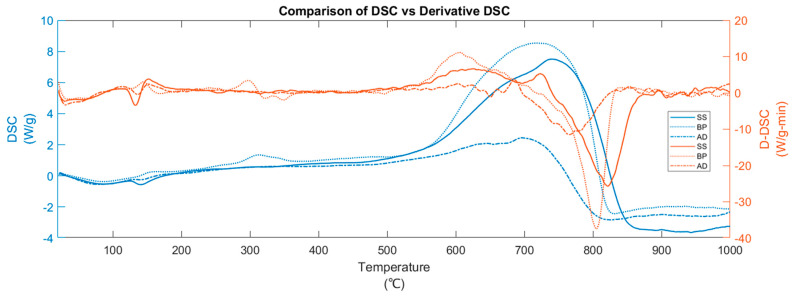
Comparison of DSC and D-DSC curves during pyrolysis of BP, SS, and AD.

Figure 4 and Figure 5, respectively, show the heat of pyrolysis (HoP) and biochar yield as well as the derivative DSC (D-DSC) and DSC curves of the three substrates during pyrolysis (at 15 °C/min). HoP is endothermic until 253, 374, and 424 °C for BP, SS, and AD, respectively. These late exothermic onsets of SS and AD are due to their extended drying stages and ash content. Higher HoP in BP also confirms the predominantly exothermic char-forming reactions seen in some biomass pyrolysis [97,98]. The active pyrolysis of AD has less HoP than SS. Although isolating and removing additives in AD feedstock can increase the heat release during pyrolysis on a laboratory scale, it will be resource-intensive on an industrial scale.

The active pyrolysis zone of BP shows two exothermic peaks in D-DSC (Figure 5) from the decomposition of xylan-form of hemicellulose [97], cellulose, and lignin with mainly CO_2_ evolution. Thus, the interactions of K and its salts may cause a shoulder peak of hemicellulose to be still visible in BP. After active pyrolysis, HoP increases owing to HeSTR and HoSTR—cracking, partial oxidation, and condensation. This is referred to as secondary pyrolysis (SP) and constitutes the rate-determining reactions until the onset of gasification [99]. At slow heating rates, this phase is influenced by the transport of evolved volatiles (with external and internal mass transfer limitations) away from the char. Secondary pyrolysis can be minimized by lowering sample mass and increasing purge rate [100]. However, this is not representative of an industrial pyrolysis process. The polycondensation of non-graphitizing carbon, such as biomass, occurs between 500 and 1000 °C. At T > 500 °C, carbon atoms form more aromatic rings. The formation of such stable sp^2^ hybridized carbon releases binding energy as heat. For oxygen-rich precursors such as biomass, distorted graphene structures (DGS) form during this stage along with regular graphene structures. These DGS are aromatic with oxygen heteroatoms and O-structures at edge carbon atoms (planar fringes) within their fjord regions. These O-structures can be readily desorbed due to their protonated form. The desorption of oxygen atoms chemisorbed on the basal plane of carbon matrix also occurs during secondary pyrolysis [101]. These O availabilities enable the precedent for partial or complete exothermic oxidation of some carbon. The loci of secondary pyrolysis and polycondensation overlap.

HoP reduces from 700, 720, and 700 °C for BP, SS, and AD, respectively, due to the competing decomposition of carbonates and inorganics, and gasification. The D-DSC curve reveals the endothermic dips from carbonates in the region of 600–700 °C for AD and 650–720 °C for SS. This agrees with the corresponding determination from FT-IR. BP lacks such a region due to low ash content. For all substrates, the next endothermic peak (around 800 °C) of D-DSC is from the formation of turbostratic carbon [102]—the DGS twists and connects to form cross-links and non-hexagonal rings (some studies refer to them as fullerene structures [103] and widely debated) form at these bridging regions effectively rendering them non-graphitizing carbon. After 800 °C, the net heat flow remains endothermic for all substrates.

After drying, the biochar yield is the lowest for BP at any given temperature due to its highest volatile matter concentration (Figure 4). At the end of active pyrolysis, yields of mineral and ash-rich biochars, SS and AD, are similar. AD has a higher yield after carbonate decomposition. The yield of mineral- and ash-rich biochar also includes silicates and/or non-volatile minerals. Thus, biochar yield is not a comparative metric for such pyrolysis.

### 3.4. Thermal Kinetics

The data used for kinetic analysis agree with the pre-calculation checks (Section S1.5 and Figures S8–S13) It is important to note that the activation energy mentioned hereon is the variable or apparent activation energies associated with solid-state reactions (Section S2.2). The E_α_ of the three investigated substrates calculated using isoconversional methods—KAS, Starink, Friedman, and NLN (Figure S14)—show varying activation energies throughout the extent of conversion (0.08 to 0.9). The methods based on linear approximations—KAS and Starink—show similar values. However, varying activation energies imply concurrent reactions, where the applicability of such linear isoconversional methods is limited. The differential method and NLN are better suited. Although the former is expected to have noise from numerical differentiation, large datasets (here, even the fastest heating rate—20 °C/min—has 3000 data points in TG) will have smoother estimates of derivative. In addition, the reactions during the slow pyrolysis of these substrates have temperature-independent reaction heats (i.e., the type of thermo-chemical transformations are invariant at these six heating rates). Thus, in agreement with the literature [104,105], NLN and differential methods result in similar E_α_. Activation energies calculated using NLN are used in further plots and calculations.

**Figure 6:**
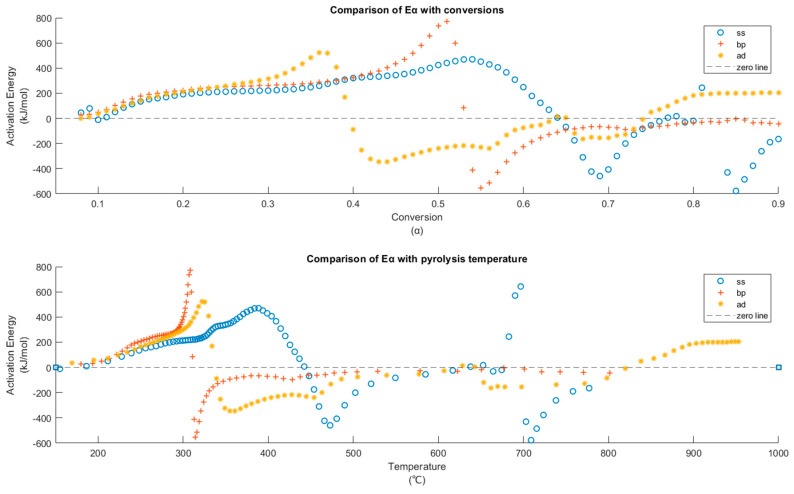
E_α_ vs. α and E_α_ vs. T_avg_ (average temperature at all heating rates) for the three substrates BP, SS and AD.

The variation of E_α_ with conversion and temperature (average temperature at all six heating rates) is shown in Figure 6. For SS, BP, and AD, E_α_ becomes negative after 0.65α, 0.54α, and 0.40α, respectively. Only AD shows a trend reversal to positive E_α_ after 0.75α. The positive E_α_ during active pyrolysis can be interpreted as follows. Here, E_α_ is primarily influenced by holocellulose and lignin. Studies have reported varying ranges of E_α_ for hemicellulose, cellulose (208 kJ/mol), and lignin (174–322 kJ/mol) for the same biomass. There are reported inconsistencies amongst these values across different investigations in the literature [16,18,106] due to variations in their chemical structure [107], the impact of AAEM, thermal lag, kinetic compensation effect, and instrumental errors. However, in these studies, the range of E_α_ always follows the trend hemicellulose > cellulose > lignin. Such a pattern is seen here for E_α_ of all the three substrates from the onset of their respective active pyrolysis and reaches a maximum at 0.54α (404 °C) for SS, 0.51α (308 °C) for BP, and 0.36α (321 °C) for AD. Until they reach this α threshold, E_α_ follows the order BP ⪆ AD ⪆ SS and is similar to that of other biomass reported in [108]. The notable variations in E_α_ with increasing conversion suggest parallel decomposition of biomass components [109] influenced by catalytic activity and secondary reactions, which is confirmed by the exothermicity during this zone (Section 3.3).

The increase in E_α_ of BP and AD, as they approach MPT, is from the start of pyrolysis of lignin and higher-order cellulose catalyzed by K and/or Na [107,110] or from aromatization of char [111]. For SS, the increase is more gradual as the decomposition shifts from polysaccharides to recalcitrant aromatics with increasing α [112]. This increasing tendency of E_α_ has also been widely reported [113,114] for other biomasses. The inorganics (such as silicates) in SS become random endothermic nucleation sites from where organic macromolecules undergo pyrolysis along a one-dimensional route. This reduces its E_α_ compared to AD (during active pyrolysis), despite having similar holocellulose content [115]. Until MPT, the active pyrolysis of BP is slowest (lowest pyrolytic reactivity due to high activation energy) [116] compared to AD and SS, which possess more inorganic species. For SS, after the MPT, the E_α_ continues to rise as with the decomposition of the aromatic lignin structures, which needs higher enthalpy of activation. However, BP and AD decline into negative values, implying an increase in exothermicity [117], which is usual in practical applications of pyrolysis [81]. By now, about 31 wt %, 30 wt %, and 24 wt % mass loss for SS, BP, and AD, respectively, have been completed. At this α, wt % loss is greater than the total volatile matter for SS, while it is lower for BP and AD. The greater fluctuation in E_α_ after active pyrolysis is evidence of varying reaction steps with temperature [118].

During active pyrolysis, at α = 0.54 (313 °C), 0.65 (448 °C), and 0.40 (339 °C) for BP, SS, and AD respectively, E_α_ transitions to negative values as -Eα. In the literature, this is generally attributed to the decrease in reaction rate with increasing temperature [119] due to complex reaction mechanisms [120]. However, explanations are scarce. Since the basic assumption of most solid-state decomposition kinetics is the Arrhenius conformity, the -E_α_ signifies non-Arrhenius behavior [121]. Usually, possibilities of other spontaneous chemical reactions and/or reaction mechanisms during -Eα are not considered when pyrolysis follows an endothermic route [122]. However, from the DSC curves (Figure 5), it was seen that the substrates follow an exothermic active and secondary pyrolysis. Another study points out that inhomogeneity present in samples such as SS leads to the crossing of wt % vs. temperature curves at different heating rates for a substrate [123]. Here, for SS, if kinetic calculations were performed with 5, 15, and 20 °C/min (three TG curves that do not cross), still, the E_α_ turns out negative after α ≈ 0.63. Hence, attributing -Eα solely to sample inhomogeneity is also inadequate. As seen in [122], for endothermic pyrolysis, a shift toward negative activation energy would mean the endpoint of volatiles decomposition with the evolution of CH_4_ and C_2_H_2_ matching the regime of -Eα. Although CH_4_ is present here, its release pattern does not match with the -Eα trend, and pyrolysis is exothermic (Figure 5). Reactions with negative activation energy are also seen in nature—ozone depletion. In terms of complex solid-state reactions such as biomass pyrolysis, there are other plausible explanations (Section S2.5) [111].

During active pyrolysis, E_α_ gradually increases with temperature/conversions for all substrates, i.e., with increasing devolatilization of organics, the rate control is shifting to alternate paths within a network of parallel reaction pathways available for the biomass components. Then, the concave transition in E_α_ to negative values hints at the shift in rate control to a reversible step within the same reaction pathway [124,125] that has a pre-equilibrium formed from an exothermic reaction. Until MPT, biomass components depolymerize endothermally (Equation (7)) to interim product (int.prod) and non-condensable gases (NC). This int.prod converts to stable char and primary tar (Tar_1_) as in Equation (8). Due to preferential char formation, the net heat release (−ΔH_0_ + ΔH_01_) is exothermic.

(7) $$\underset{\left(1-w\right)}{Biomass\;Component}\;\underset{-\Delta H_{0}}{\overset{k_{0}}{\to }}Int.Prod+NC\left(g\right)$$

(8) $$Int.Prod\underset{\Delta H_{01}}{\overset{k_{01}}{\to }}Char+Tar_{1}\;\left(g\right)$$

(9) $$Int.Product+\underset{\left(1-\phi \right)}{L}\;\overset{k_{1}}{\underset{k_{2}}{⇌}}\underset{a^{*}}{Char^{*}}+\underset{\phi }{L\left(Tar_{2}\right)}$$

(10) $${\underset{a^{*}}{Char}}^{*}+\underset{\phi }{L\left(Tar_{2}\right)}\overset{k_{d}}{\underset{k_{a}}{⇌}}\;\underset{1-\phi }{L}+\underset{P}{Tar_{2}\left(g\right)+Char}\;$$

(11) $$Tar_{2}\left(g\right)\;\overset{k_{3}}{\to }\;\mathrm{Sec}\mathrm{ondary}\;Char+NC\left(g\right)\;$$

(12) $$M_{x}CO_{3}\;\overset{k_{4}}{\to }\;M_{x}O+CO_{2}$$

After MPT, int.prod is formed from higher ordered cellulose and more stable organics such as lignin. Then, as in Equation (9), int.prod decomposes to porous metastable amorphous char*. The heavy secondary tar (*Tar*_2_) desorb out of the pores, and char undergoes an exothermic structural change to a more stabilized form. Here, L is an active site, *L*(*tar*_2_) is the active site filled with heavy tar vapor molecule, *ϕ* is the fraction of active sites filled with *Tar*_2_, (1 − *ϕ*) is the fraction of empty active sites, and a* is the activity of the metastable char*. Based on the microscopic reversibility principle (i.e., the rate of each elementary step is equal to rate of its reverse process) r_1_ = r_d_ = 0 at equilibrium conditions (*P* = *P_eq_* and *ϕ* = *ϕ_eq_*) [126]. Then, *P_eq_* is given by Equation (15).

(13) $$r_{1}=k_{1}\left(1-\phi \right)-k_{2}\phi a^{*}$$

(14) $$r_{d}=k_{d}a^{*}\phi -k_{a}\left(1-\phi \right)P\;$$

(15) $$P_{eq}=\frac{k_{1}k_{d}}{k_{2}k_{a}}=K_{1}K_{d}\;$$

(16) $$a^{*}=\exp\left(\frac{\Delta G^{*}}{RT}\right)$$

where *K*_1_ and *K_d_* are thermodynamic equilibrium constants for decomposition and desorption, respectively, and are given by the Van Hoff’s Equations (17) and (18).

(17) $$K_{1}=A_{1}\exp\left(-\frac{\Delta H_{1}}{RT}\right)=\exp\left(\frac{\Delta S_{1}}{R}\right)\exp\left(-\frac{\Delta H_{1}}{RT}\right)$$

(18) $$K_{2}=A_{2}\exp\left(-\frac{\Delta H_{d}}{RT}\right)=\exp\left(\frac{\Delta S_{d}}{R}\right)\exp\left(-\frac{\Delta H_{d}}{RT}\right)$$

where Δ*H*_1_ and Δ*H_d_* are standard enthalpy changes, and Δ*S*_1_ and Δ*S_d_* are standard entropy changes associated with decomposition and desorption, respectively. By pseudo steady-state hypothesis, the reaction intermediates remain constant with time, i.e., r_1_ = *r_d_*. If desorption is the rate-limiting step with k_d_a*, *k_a_P* ≪ *k*_1_, *k*_2_a* then:

(19) $$\phi =\frac{k_{1}}{k_{1}+k_{2}a^{*}}=\frac{1}{1+\frac{a^{*}}{K_{1}}}=\frac{1}{1+\exp\left(\frac{\Delta G^{*}+\Delta G_{1}}{RT}\right)}$$

where ΔG* is the positive free energy of formation of the metastable char by release of ΔH^*^. The reaction rate will then be equal to the rate of desorption, the rate-limiting step, *r* = *r_d_* (Equation (20)).

(20) $$r_{d}=k_{d}\left(a^{*}\phi -\frac{k_{a}}{k_{d}}\;\left(1-\phi \right)\;\right)$$

(21) $$=k_{d}a^{*}\left(\phi -\frac{K_{1}}{a^{*}}\left(1-\phi \right)\frac{P}{P_{eq}}\right)$$

(22) $$r_{d}=k_{d}a^{*}\phi \left(1-\frac{P}{P_{eq}}\right)\;∵\frac{a^{*}}{K_{1}}=\frac{1-\phi }{\phi }$$

(23) $$r_{d}=A_{d}A^{*}\exp\left(-\frac{\left(E_{d}-\Delta H^{*}\right)}{RT}\right)\;$$

When pressure gradients within the experiment are considered negligible and when ϕ ≈ 1 (all active sites are filled with tar_2_), Equation (22) becomes Equation (23). Here, ϕ ≈ 1 when either (a) *RT* ≫ (ΔG* + Δ*G*_1_), i.e., at higher temperatures after MPT, and (b) Δ*G*_1_ ≪ −ΔG*, i.e., when decomposition is spontaneous due to the exothermic char formation reactions as seen in DSC. Thus, when the rate control shifts to reaction r_d_, we obtain negative activation energies. After this transition to negative values, E_α_ has a general increasing trend with conversion for all the three investigated substrates. It means that reactions again shift to alternate pathways among a network of available pathways. This point of lowest -Eα from which apparent activation energy starts again increasing marks the start of the increasing influence of HeSTR (resulting in secondary char formation) due to longer contact times with the biochar (Equation (21)). An increase in exothermicity (as seen in DSC) and the presence of non-condensable gases corroborate this observation. Lower bulk density increases HeSTR [127] as it hinders the release of tar vapors and improves aromatization [128]. This is because at lower bulk densities, the height of the substrate increases (diameter of crucible being a constant), which increases the residence time of tar in the matrix [129]. In this case, the start of HeSTR for the substrates follows the order of their increasing bulk densities—BP < AD < SS. HeSTR can also be influenced by the catalytic effect of AAEM in the three substrates [3].

The temperatures between 650 and 700 °C for SS and those above 800 °C for AD shift toward positive E_a_ due to the change in rate control to reactions of carbonate decomposition (Equation (22)). After this, -E_α_ arises from the gasification of char and its associated external diffusion limitations. At these temperatures, further interpretations of observed kinetic parameters would be incomplete as, even at particle sizes below 0.5 mm, these regions would deviate away from the pure kinetic regime depending on the Biot and Pyrolysis (thermal Thiele modulus^−1^) numbers [81]. Temperatures between 500 and 700 °C also are dominated by HoSTR, the lateral growth of aromatic chains, and crosslinking [81], as seen from the DSC exotherm until around 800 °C beyond which reaction control shifts to endothermic graphitization, inorganic decomposition, and high-temperature gasification.

#### 3.4.1. Pre-Exponential Factor (A)

When the frequency factors are calculated based on pseudo KCE (pKCE), the values of ln A should change proportional to E_α_ (Section S2.4); the collision probability of molecules decreases with an increase in temperature reducing the reaction rate [111]. Processes such as surface reactions, diffusion, adsorption, etc. usually have A < 10^9^ s^−1^. However, Table 2 shows the poor linearity (R^2^ < 0.99) between ln A and activation energy. From the review of the conditions that lead to a pKCE, this conclusion becomes apparent. The pKCE arises from the propagation of systematic and computational errors during kinetic measurements and calculation [130] and does not have a physical origin like the true KCE [131]. Hence, a weak pKCE implies that the temperature ranges over which the kinetic analysis was performed are sufficient, and this consequently led to fewer errors in E_α_ estimation [132,133]. Thus, the calculation of A based on pKCE as seen in other investigations is not viable here.

#### 3.4.2. Enthalpy

**Figure 7:**
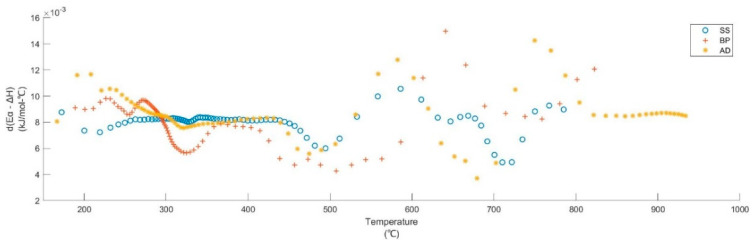
Rate of change of potential barrier during pyrolysis of BP, SS, and AD.

**Figure 8:**
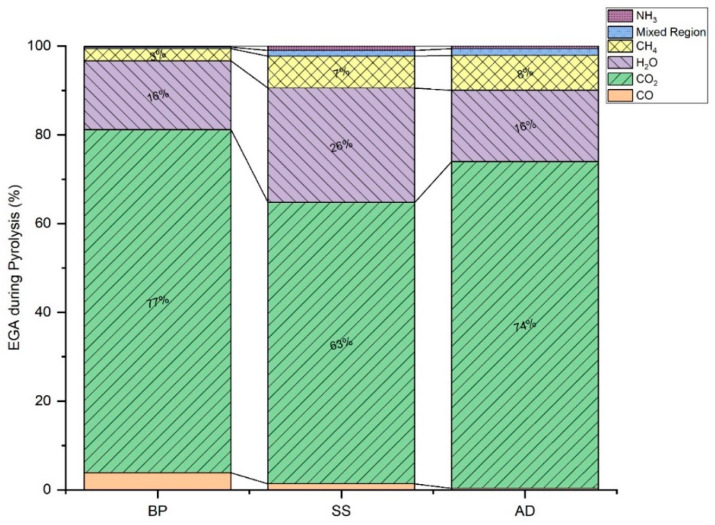
Normalized evolved gas analysis (EGA) during the pyrolysis of BP, SS and AD with HTT 745, 735, and 690 °C, respectively.

The enthalpy variation also follows the same trend as E_α_ as they are calculated from Eyring equations (Section S2.6). In many kinetic investigations [134,135,136,137], the active pyrolysis of biomass is reported as a net endothermic reaction (with minor exothermic peaks, if any, caused by oxidative micro-environments arising from the evolved gases) due to the heat requirement for macromolecular devolatilization. Here, during the active pyrolysis of the three substrates, ΔH is positive, and the DSC curves indicate that the reaction tends to be exothermal until temperatures exceed 700 °C. This is further evidence of exothermic char formation and HeSTR that are present throughout the active and secondary pyrolysis regions, respectively. Similar cases have been reported for other biomasses [138,139,140]. The smaller the (E_α_ − ΔH_α_) at any conversion, the more favorable the product of formation [139,140,141] due to the lower potential barrier. It increases with conversion for all three substrates, signifying the expected slowdown of pyrolysis due to the exhaustion of organic matter and changes in reaction mechanisms. The d(E_α_ − ΔH_α_), in Figure 7, shows the rate of change in the potential barrier of pyrolysis with temperature. The last exothermal peak of DSC occurs at around 700, 720, and 700 °C for BP, SS, and AD, respectively. After this, (a) the HeSTR and HoSTR slow down with an increase in temperature, and (b) inorganic calcium and magnesium carbonates are also completely decomposed (also observed in the DTG curve—Figure 2). This is an agreement with the d(E_α_ − ΔH_α_). The process slows down considerably after ≈745, 735, and 690 °C for BP, SS, and AD beyond, and there is also a large increase in CO and CO_2_ emissions. These MWBs favor exothermic char formation during active pyrolysis and HeSTR during secondary pyrolysis. It is also in agreement with the findings that biomass pyrolysis can conditionally favor exothermic [4,142,143,144] or endothermic [122,136,137,145,146] behavior. The various reaction regimes and the normalized emission of gases during the pyrolysis of these substrates are summarized in Table 3 and Figure 8, respectively.

## 4. Conclusions

The pyrolysis of conventional lignocellulosic biomass is widely investigated for energetic and value-added product applications. The MWB results from anthropogenic activities such as wastewater treatment and chemical fertilization, and it has limited recyclability owing to the high AAEM, silicate, ash, and/or heavy metal content. Their transformation to biochar presents a feasible route for their sustainable recycling in the field of solid waste management. Three different types of MWB—BP, SS, and AD—are investigated to elucidate their thermolysis mechanism and showcase the potential for biochar synthesis in a TGA scale. The biochar synthesis favors char formation kinetics and is exothermic in the order BP > SS > AD. Exothermicity can be maximized without increasing residence time and GHG (CO and CO_2_) emissions if the pyrolysis is performed until the HTT of 745, 735, and 690 °C for BP, SS, and AD. Here, mineral-rich and ash-low BP yields the most HoP, while SS and AD also evolve 7–8% CH_4_ for energy recovery. GHG emissions of CO_2_ and CO are the highest for BP (77% and 3.83%, respectively). Thus, there is a potential for further optimizing biochar synthesis for energy recovery and GHG emissions through the combined pyrolysis of MWB and warrants further investigations. The HeSTR is prominent from ≈318, 481, and 376 °C for BP, SS, and AD, respectively, while HoSTR is from ≈565 °C. The endothermic gasification becomes rate-limiting from 800 °C.

It can also be concluded that:

1. The pyrolysis of BP has the highest heat release and most kinetic favorability. Its biochar yield is the smallest due to high cellulose and low silicate content.
2. Amongst the various stages, the highest contribution of exothermicity is from secondary pyrolysis and emission of NO_x_ precursors follows the order SS > AD > BP.
3. Based on isoconversional methods, negative apparent activation energies are intrinsic to explain their kinetics after 313, 448, and 339 °C for BP, SS, and AD, respectively.

These results can support modeling and controlling biochar synthesis from the slow pyrolysis of MWB sustainably by maximizing heat uptake and minimizing GHG emissions. Such aspects are presently not addressed in the present biochar literature. Furthermore, the detailed methodology applied in this work can be utilized to critically analyze the pyrolysis schemes of other MWBs and their blends. The investigations of physio-chemical attributes of the MWB-derived biochar and the influence of process scaling can complement these results in tailor-made biochar synthesis for the development of their pyrolysis technology. Hence, future studies in the direction of process scale-up, biochar assay, and life cycle assessment are recommended. In the follow-up part-2 publication, the authors explore the properties of biochar for its application in carbon sequestration, adsorption and soil application.

## Figures and Tables

**Table 1:** Wavelength assignment for chemigrams of online FT-IR.

Evolved Gases	Wavelength (cm^−1^)
CO_2_	2400–2250
CO	2250–2000
H_2_O	3990–3400
CH_4_	3020–2800
Mixed Region	1200–1000
NH_3_	980–920

**Table 2:** Linear relationship (pseudo KCE) between ln A and E.

Sample	Conversions	R^2^ of Pseudo KCE at Different Heating Rates					
	α	5 K/min	7 K/min	10 K/min	12 K/min	15 K/min	20 K/min
BP	0.08 to 0.53	0.943	0.971	0.962	0.963	0.930	0.952
SS	0.12 to 0.64	0.885	0.823	0.902	0.900	0.893	0.885
AD	0.08 to 0.39	0.849	0.895	0.894	0.826	0.793	0.763

**Table 3:** Thermal regions during the slow pyrolysis of BP, SS, and AD between 25 and 1000 °C.

Substrate	Temperatures of Interest	Predominant Reactions	Weight Loss %	Heat of Pyrolysis	Reaction Progress	MPT	DTG	Predominant Evolved Gases
	°C		%	kJ/kg	%	°C	%/min	
BP	45–119	Drying	3.95	−99.49	5.10	76.61	−1.06	H_2_O
	156–519	Active pyrolysis	57.24	1172.9	73.83	304.32	−10.93	CO_2_, CO, CH_4_, H_2_O, volatiles
	519–699	Secondary Pyrolysis	3.97	3237.8	5.12	NA	−0.49	CO, CO_2_, CH_4_
	699–744	Carbonate decomposition + secondary pyrolysis	1.68	1529.5	2.16	724.23	−0.59	CO, CO_2_
	869–1000	Other inorganic decomposition + gasification	5.79	−1087.2	7.48	995.88	−0.73	CO, CO_2_
SS	49–120	Drying	3.64	−152.8	6.70	75.08	−1.00	H_2_O
	122–145	Extended drying	1.21	−50.7	2.23	134.41	−1.18	H_2_O
	221–510	Active pyrolysis	32.57	663.2	59.84	329.90	−3.57	CO_2_, CH_4_, H_2_O, NH_3_
	510–644	Secondary pyrolysis	2.64	1306.1	4.85	NA	−0.49	CO_2_, H_2_O
	644–737	Carbonate decomposition + Secondary pyrolysis	5.76	2300.2	10.59	709.23	−1.36	CO, CO_2_
	737–1000	Gasification	6.65	−440.6	12.21	809.09	−0.49	CO, CO_2_, CH_4_
AD	37–120	Drying	5.06	−197.0	7.93	66.35	−1.22	H_2_O
	196–499	Active pyrolysis	31.03	432.1	48.58	307.75	−3.25	CO_2_, CH_4_, H_2_O
	499–629	Secondary pyrolysis	2.73	582.6	4.28	NA	−0.49	CO_2_, H_2_O
	629–690	Carbonate decomposition + Secondary pyrolysis	3.69	469.0	5.77	669.59	−1.20	CO, CO_2_
	821–1000	Other inorganic decomposition + gasification	16.30	−1964.1	25.52	968.93	−2.32	CO, CO_2_

## Data Availability

Available upon request.

## Supplementary Materials

The following supporting information can be downloaded at: https://www.mdpi.com/article/10.3390/ma15124130/s1. Supporting results and theory used for calculation are detailed in the Supplemental Material [16,26,34,51,109,121,147,148,149,150,151,152,153,154,155,156,157,158,159,160,161,162,163,164,165,166,167,168,169,170].

## Abbreviations

AD	Anaerobic digestate
A	Pre-exponential factor corresponding to E_α_
BP	Banana peduncles
D-DSC	Derivative differential scanning calorimetry
DSC	Differential scanning calorimetry
DTG	Differential thermogravimetry
E_α_	Apparent activation energy at conversion α
FT-IR	Fourier transform infrared spectroscopy
HeSTR	Heterogenous secondary tar reactions
HHV	Higher heating value
HoP	Heat of pyrolysis
HoSTR	Homogenous secondary tar reactions
HTT	Highest treatment temperature
KCE	Kinetic compensation effect
MPT	Maximum peak temperature
MWB	Mineral- and ash-rich waste biomass
NC	Non condensable gases
pKCE	Pseudo kinetic compensation effect
SS	Sewage sludge
TGA	Thermogravimetric analyzer
α	Conversion
