# Supplementary material for: Biochar Synthesis from Mineral- and Ash-Rich Waste Biomass, Part 1: Investigation of Thermal Decomposition Mechanism during Slow Pyrolysis
Source: Materials (Basel). 2022 Jun 10;15(12):4130. doi: 10.3390/ma15124130 (PMC9227128; doi:10.3390/ma15124130)
Supplement: Supplementary file 1 [file materials-15-04130-s001.zip › materials-1726763-supplementary.pdf]

Supplemental Material

Main Article Title: Biochar Synthesis from Mineral- and Ash-rich Waste Biomass. Part 1: Investigation of Thermal Decomposition Mechanism during Slow Pyrolysis

Authors: Rahul Ramesh Nair[1], Moni Mohan Mondal[1], Shanmugham Venkatachalam Srinivasan[2], Dirk Weichgrebe[1]

[1] Institute of Sanitary Engineering and Waste Management, Leibniz University of Hannover
[2] Environmental Engineering Department, Central Leather Research Institute

Contents

1. Additional Results 2

1.1 Proximate and elemental analysis of biomass 2

1.2 Statistics of TGA Data 3

1.3 Closed-lid TGA experiments 3

1.4 Emission Analysis 8

1.5 Checks on kinetic measurements 9

1.6 Comparison of Eα calculated using isoconversional methods 13

2. Theory 13

2.1 Pyrolysis stages 13

2.2 Apparent activation energy 14

2.3 Isoconversional Methods for Apparent activation energy 14

2.4 Preexponential factor 18

2.5 Negative Apparent Activation Energy 18

2.6 Thermodynamic Properties 19

1. Additional Results

1.1 Proximate and elemental analysis of biomass

Table S1: proximate and fiber analysis of pyrolysis feedstock - SS (sewage sludge), BP (banana peduncles) and AD (Anaerobic digestate)

#	Unit*	SS	BP	AD	Equipment/Model
P	wt.-%	1.9	0.29	2.69	ICP-OES/ SPECTROBLUE TI
K	wt.-%	0.19	6.53	4.56	
Ca	wt.-%	7.49	0.89	5.39	
Mg	wt.-%	1.25	0.2	1.4	
Na	wt.-%	0.27	< 0.0097**	5.35	
S	wt.-%	1.51	0.12	0.6	
C	wt.-%	28.5	41.9	27.4	Elemental Analyzer/ TruSpec CHN from Leco Instrumente
H	wt.-%	4.5	5.6	4.6	
N	wt.-%	2.7	0.98	2.6	
O	wt.-%	19.3	15.2	18.1	
O/C	ratio	0.404	0.225	0.376	
H/C	ratio	1.673	1.517	1.772	
As	mg/kg	4.33	< 0.1	0.6	ICP-MS/ICAP Q from Thermo Fisher
Hg	mg/kg	4.61	< 0.01	0.47	AAS/ Hydra AA from Teledyne Leeman Labs Inc
Cl	mg/kg	< 1884	4642	76815	Titration/ Metrohm Robotic Titriosampler (855) with Ag-Electrode
Pb	mg/kg	41.3	< 1.93	6.6	
Cd	mg/kg	2.12	< 0.1	0.37	
Ni	mg/kg	67.6	<1.93	18.5	
Zn	mg/kg	1703	14.6	191	ICP-OES/ SPECTROBLUE TI
Fe	mg/kg	11600	30.3	5522	
Cu	mg/kg	235	< 1.93	42.6	
Cr	mg/kg	277	2.96	38.4	
Si	mg/kg	91891	5568	42050	
Lignin	wt.-%	13.4	7.7	9.6	Muffle Furnace/ FT12 from Gerhardt
Cellulose	wt.-%	4	44.7	5.9	
Hemi cellulose	wt.-%	5.3	12.1	8	
VM***	wt.-%	27.1	65.3	42.6	Muffle Furnace

Ash Content	wt.-%	67.2	13.8	41.8	
Fixed Carbon	wt.-%	0.6	16.8	7.4	

1.2 Statistics of TGA Data

Table S2: Standard deviations of weight and heat flow (HF) measurements for 3 trials of each feedstock

Sample	Std Deviation of Weight Measurements (mg)		Std Deviation of HF Measurements (mW/mg)	
	Mean	*Maximum*	*Mean*	*Maximum*
BP	1.12	3.88	2.62	5.63
SS	1.37	2.19	1.34	4.22
AD	1.41	2.67	0.41	1.44

1.3 Closed-lid TGA experiments

For BP, SS and AD, the results of the closed-lid and open-lid TGA experiments at the heating of rate 15 ℃/min and their respective online FT-IR measurements are shown in Figures S1 to S6.

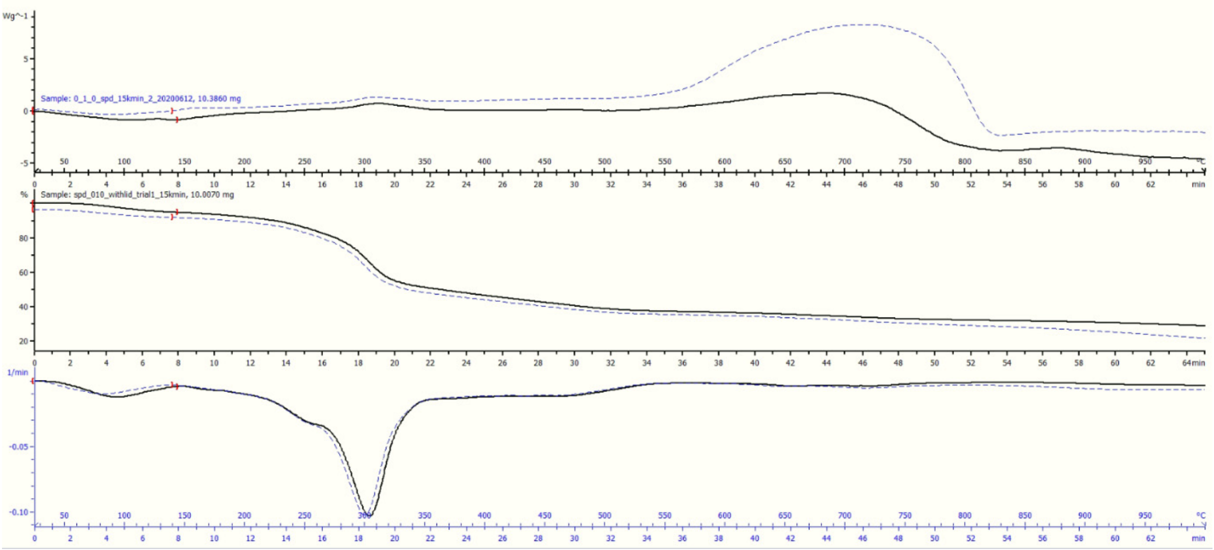

Figure S1: The DSC (top), TG(middle), and DTG (bottom) thermograms during pyrolysis of BP with (-) and without (- -) lid (plotted Mettler Toledo StarE software)

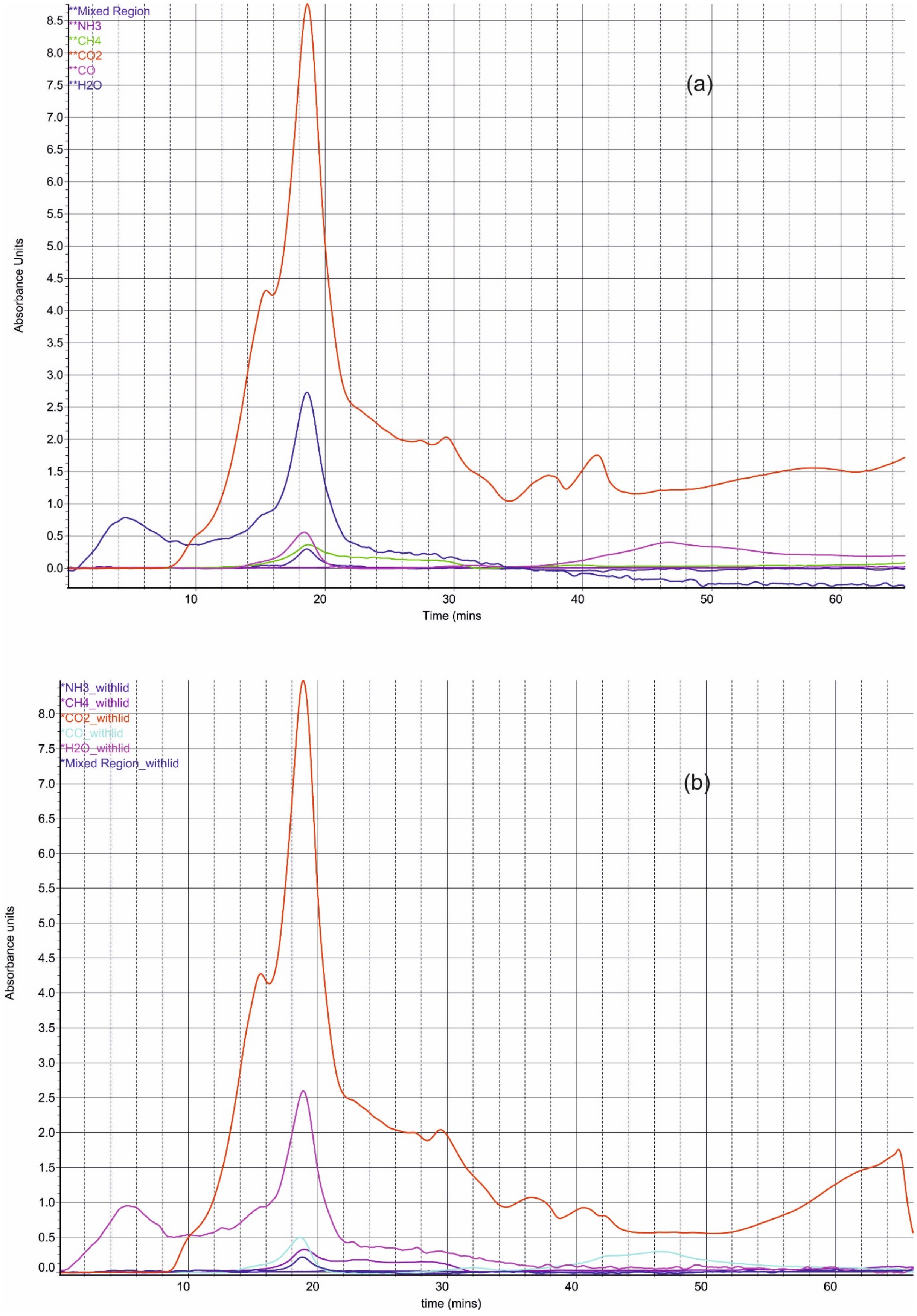

Figure S2: FT-IR chemigrams during pyrolysis of a) BP without lid and b) BP with lid

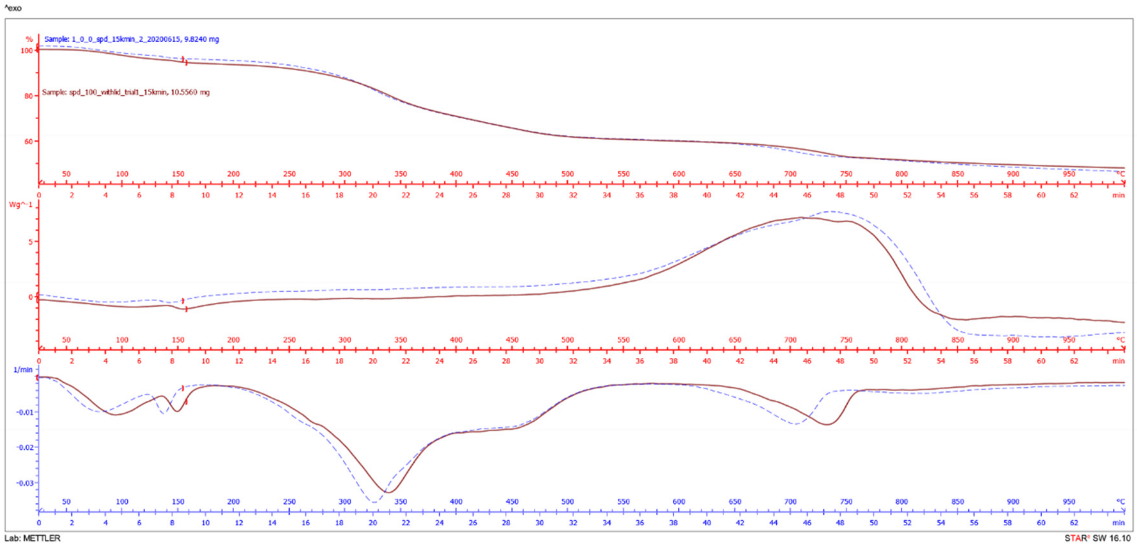

Figure S3: The TG (top), DTG (middle) and DSC (bottom) thermograms during pyrolysis of SS with (-) and without (- -) lid

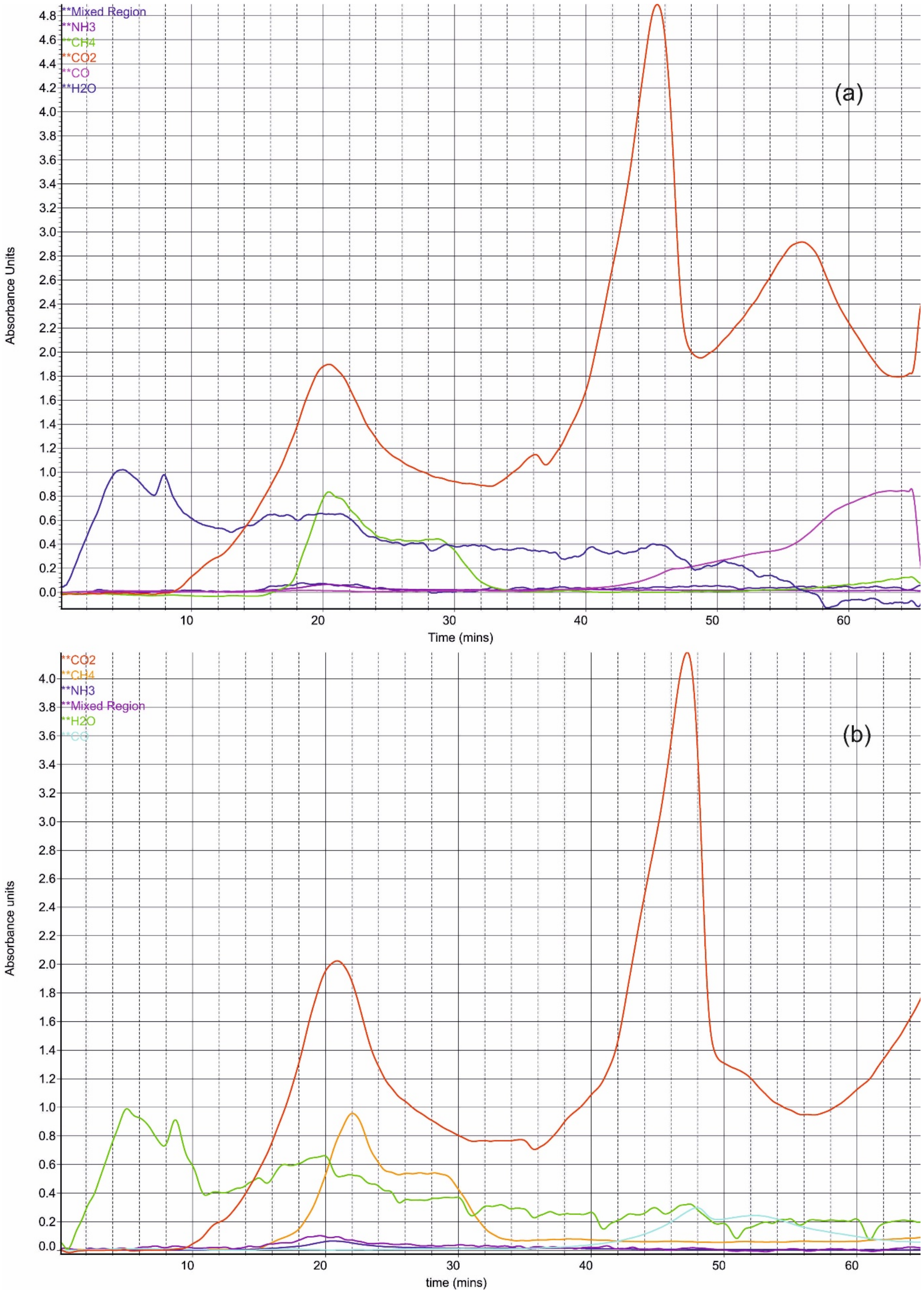

Figure S4: FT-IR chemigrams during pyrolysis of a) SS without lid and b) SS with lid

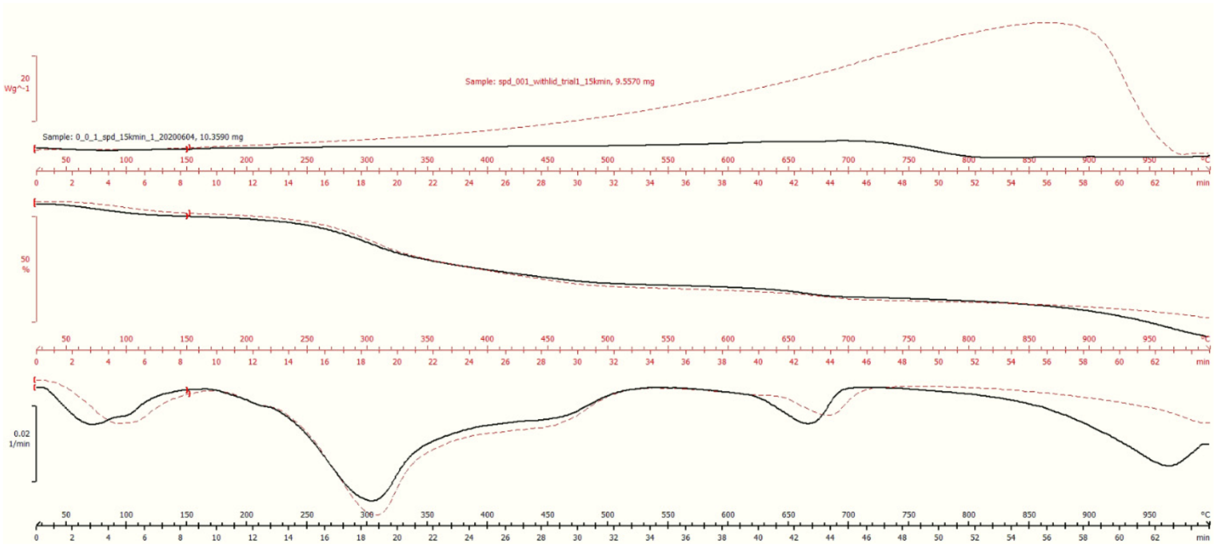

Figure S5: The DSC (top), TG(middle), and DTG (middle) thermograms during pyrolysis of AD with (- -) and without (-) lid

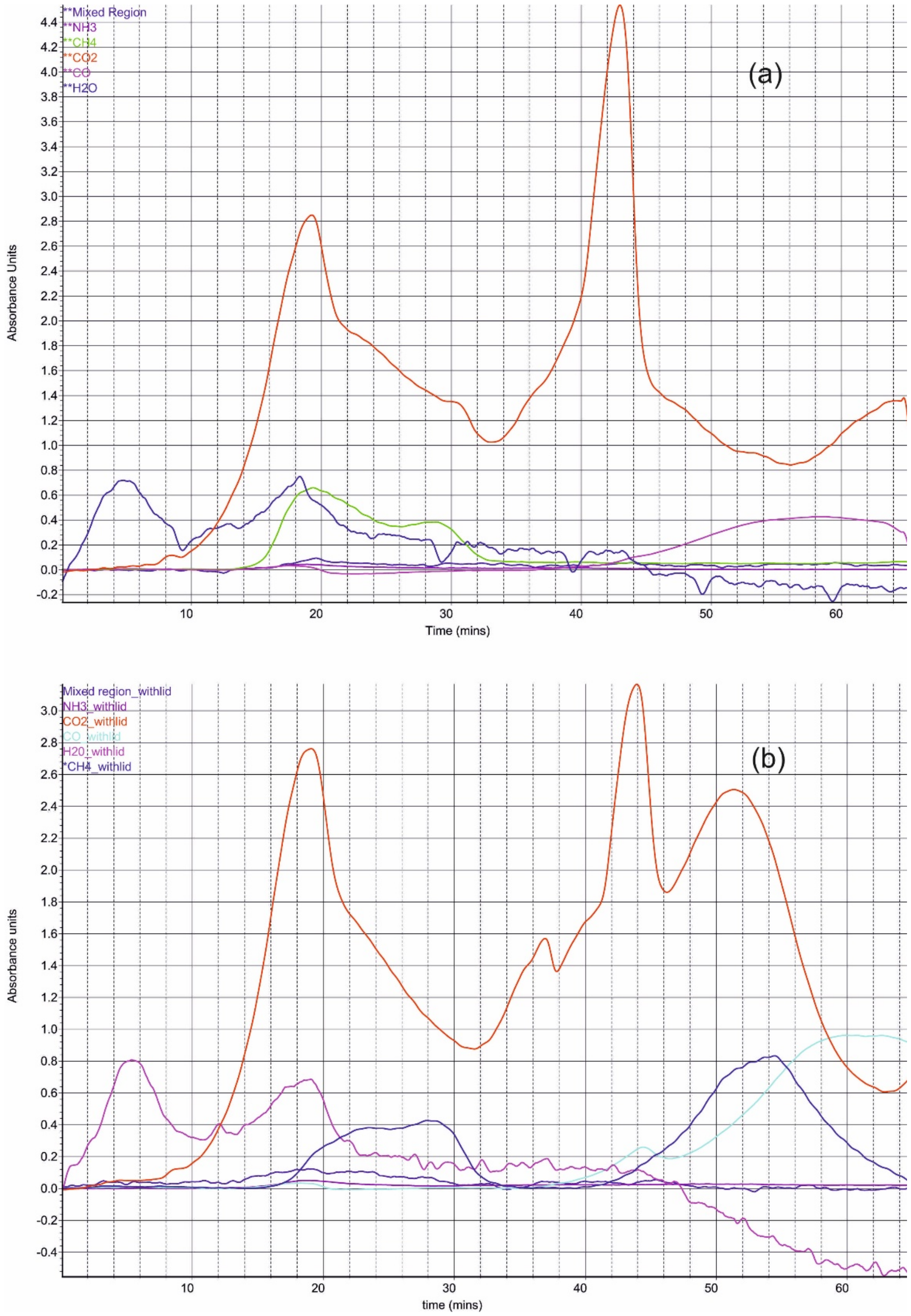

Figure S6: FT-IR chemigrams during pyrolysis of a) AD without lid and b) AD with lid

1.4 Emission Analysis

The Figure S7 denotes the NO_x precursor emissions during the pyrolysis of BP, SS and AD as measured in the FT-IR.

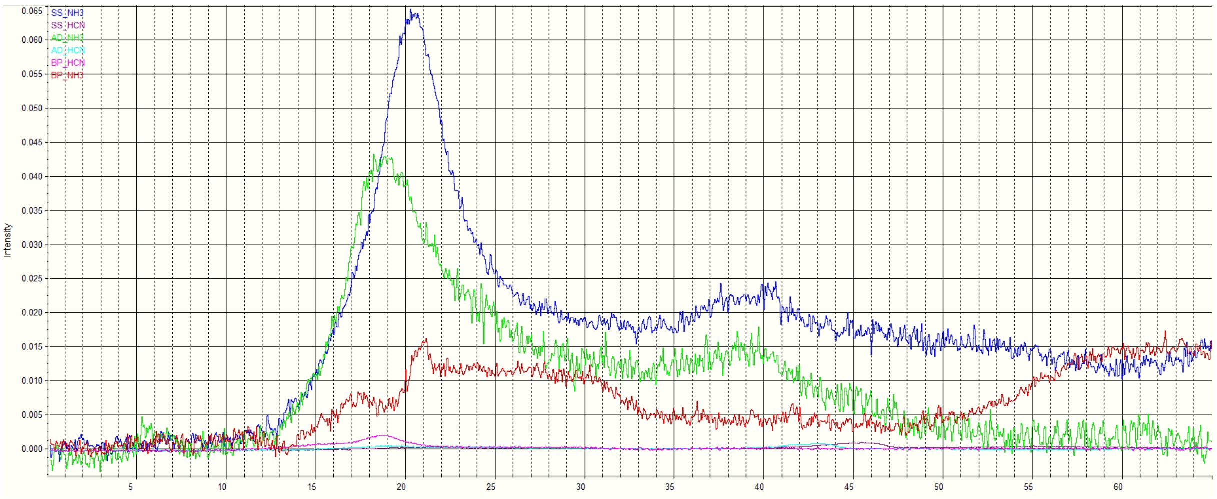

Figure S7: NOx precursor release pattern as FT-IR chemigrams during the pyrolysis of BP, SS and AD

1.5 Checks on kinetic measurements

The figures S8 – S10 show the variation in sample temperature (T_s) and reference temperature (T_r) for the three substrates at six different heating rates used for the kinetic analysis. The three substrates maintain linearity in their temperature program. The deviation of sample temperature (T_s) from the reference temperature (T_r) is only within ± 5 ℃, which is unavoidable [1] due to the thermal conductivity of the substrates. The TG instrument measures T_s at the bottom of the pan. Small variations between T_s and T_r imply minimal self-heating and thermal lag. The figures S11 – S3 indicate the profile of conversion (α) vs T_s and T_r for the three substrates at these heating rates. These plots of convey the negligible influence of thermal lag on conversions (reaction mechanisms). Hence, erroneous interpretation of kinetics data (at the six selected heating rates) due to temperature errors [2] and from heat transfer limitations is unlikely.

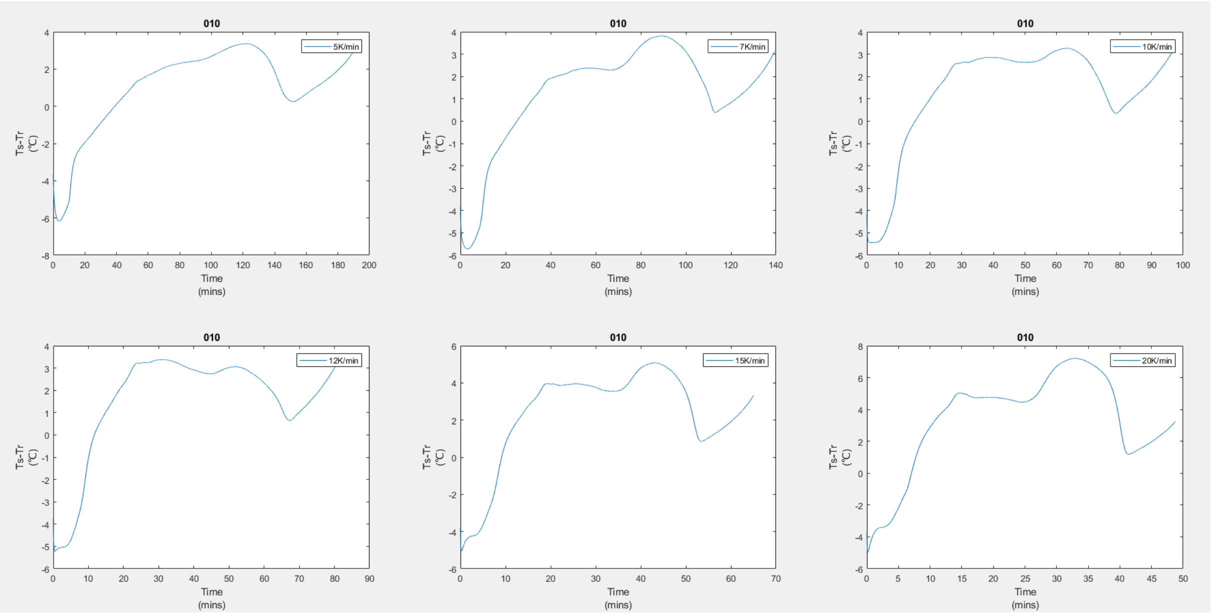

Figure S8: T_s - T_r vs time at six different heating rates for the pyrolysis of BP

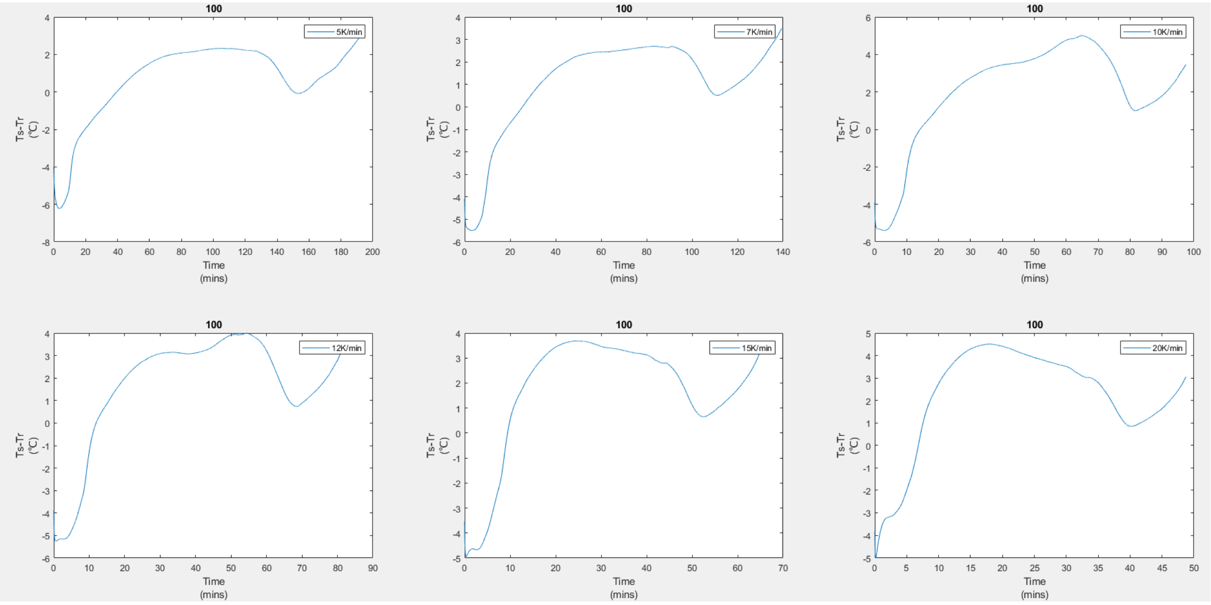

Figure S9: Ts - Tr vs time at six different heating rates for the pyrolysis of SS

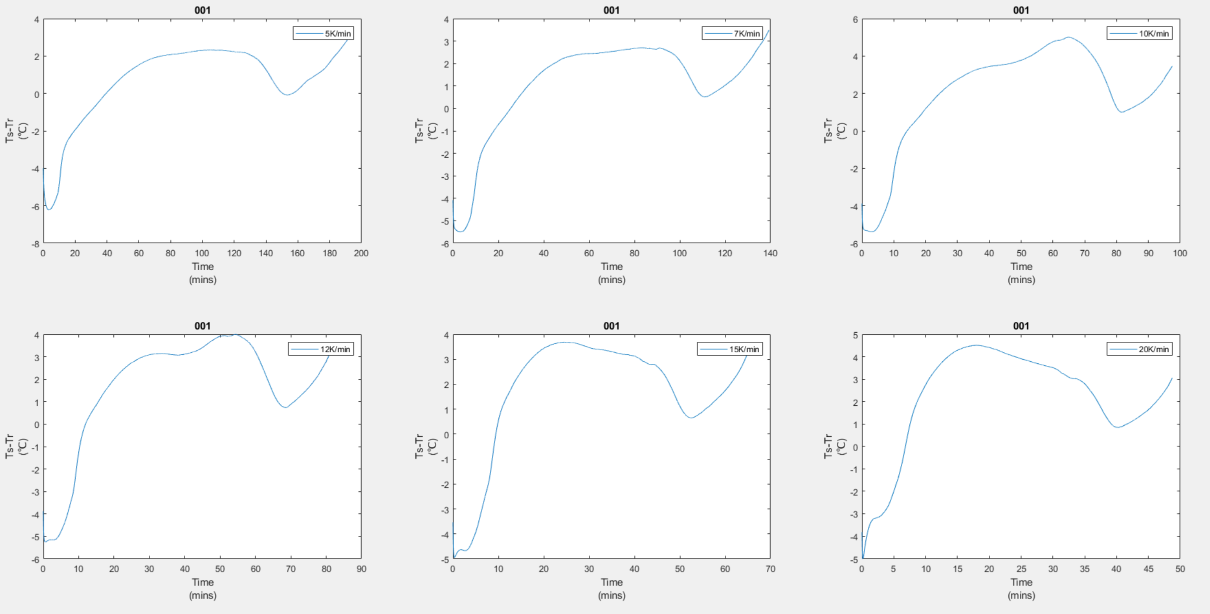

Figure S10: Ts – Tr vs time at six different heating rates for the pyrolysis of AD

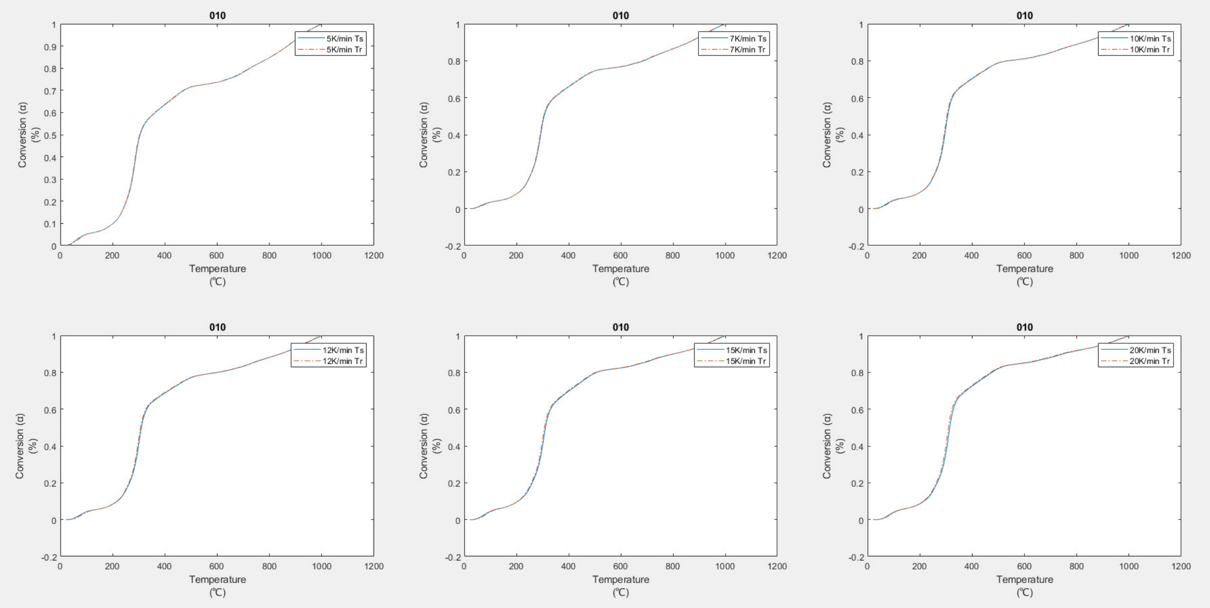

Figure S11: α vs T_s and T_r for BP pyrolysis at six different heating rates

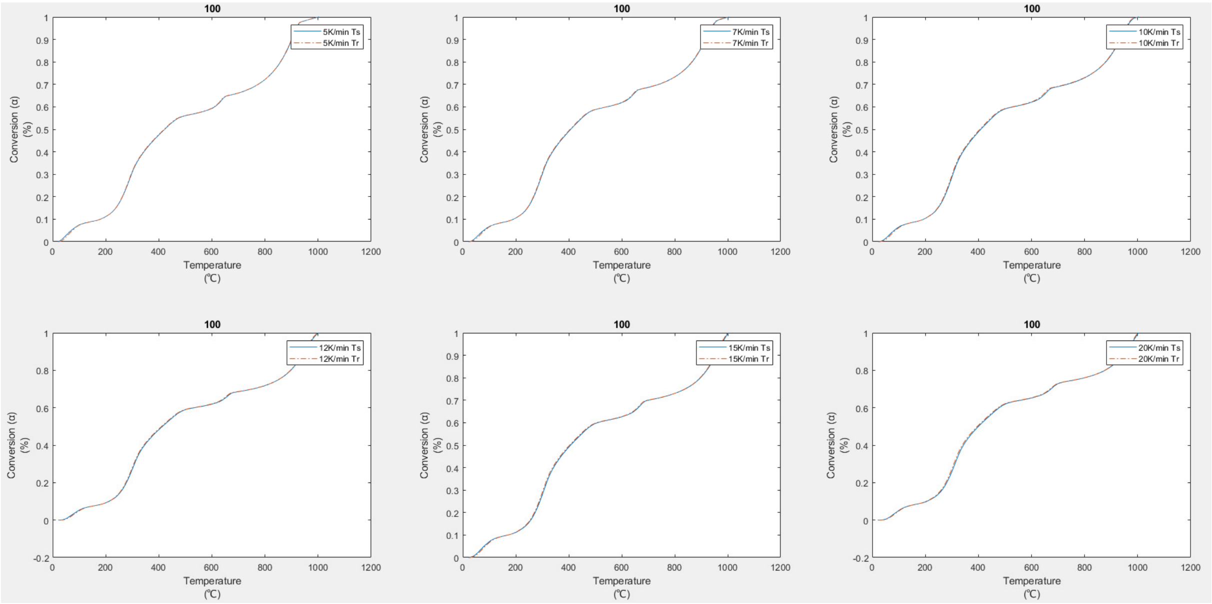

Figure S12: α vs Ts and Tr for SS pyrolysis at six different heating rates

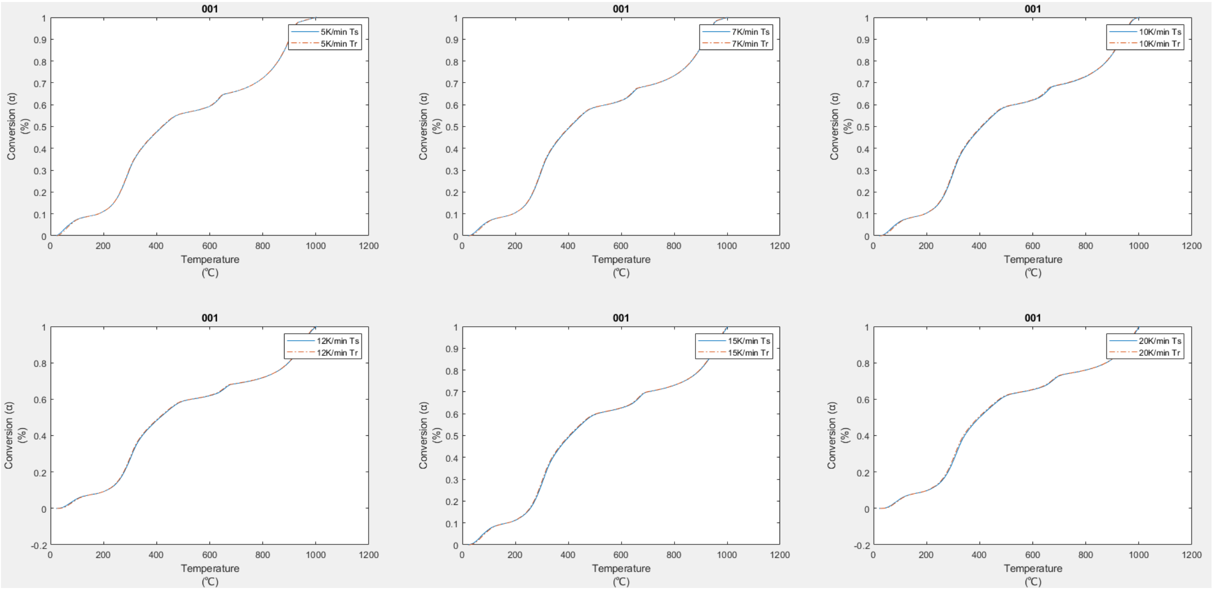

Figure S13: α vs Ts and T_r for AD pyrolysis at six different heating rates

1.6 Comparison of Eα calculated using isoconversional methods

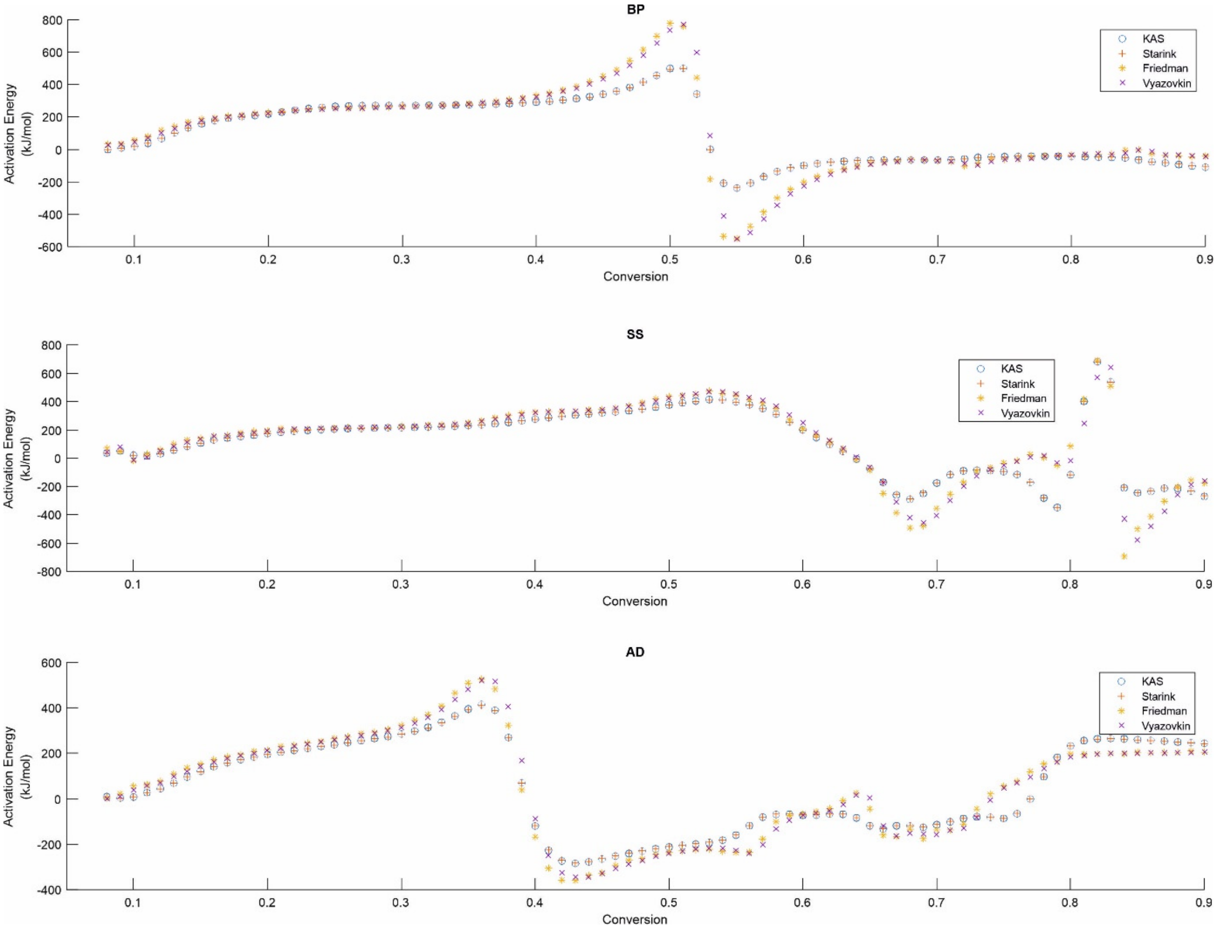

Figure S14: E_α calculated from different isoconversional methods – KAS, Starink, Friedman and NLN (Vyazovkin) for BP, SS, and AD

2. Theory

2.1 Pyrolysis stages

The general stages of biomass pyrolysis can be explained as follows. After elimination of capillary and free water, pyrolytic drying (until 150 ℃) removes chemically bound water, if present. Then, depending on the type of biomass and thermal program [1], devolatilization of lignocellulosic material proceeds through active pyrolysis. Here, tar (oxygenated compounds in vapor phase), char, and non-condensable gases are formed. After the maximum peak temperature (MPT), the autocatalytic heterogenous secondary tar reactions (HeSTR) begin with the tar volatiles in vapor phase reacting at the pore walls of the char matrix (due to higher residence time in the biochar matrix) to mainly form char, secondary tar and non-condensable gases (NC). Degradation of crystalline portion of cellulose and recalcitrant lignin also occurs above MPT until ~900 ℃, albeit at a slower rate [3]. The region above 500 ℃ is also characterized by vapor-phase cracking of tar volatiles labelled as the

homogenous (vapor phase) secondary tar reactions (HoSTR) resulting in the release of more NC [4]. HeSTR and HoSTR have a lower rate of devolatilization compared to active pyrolysis. From 500 – 1000 ℃, the aromatic condensation of char also ensues through the breaking of C-H and C-O bonds [5,6]. Identification of these different regions for a material aid in designing their pyrolysis systems.

2.2 Apparent activation energy

It is the effective value of activation energies of all the individual steps in a multi-step reaction [7]. This is different from the activation energy of a rate determining step in a single-step elementary reaction. If a pressure-independent reaction has *n* parallel steps, then the reaction rate is given as eqn 1.

$$\frac{d\alpha}{dt} = \sum_{i}^{n} k_i(T) f_i(\alpha) \tag{1}$$

$$\ln \sum_{i}^{n} k_i = \ln k_{eff} = lnA - \frac{E_{eff}}{R} * \frac{1}{T} \tag{2}$$

$$E_{eff} = -R\left(\frac{d\, lnk_{eff}}{dT^{-1}}\right) = -R\left(\frac{\delta\left(\frac{d\alpha}{dt} \times \frac{1}{f(\alpha)}\right)}{\delta T^{-1}}\right) \tag{3}$$

Using Arrhenius expression for the effective rate constant, k_{eff}, the apparent activation energy, E_{eff}, is given by the net slope of the n linear equations as shown in eqn 3. In this paper, we consider activation energy as this effective value. For isoconversional methods, f(α) is considered a constant and hence eqn 3 becomes eqn 4 where E_α is the effective of all the activation energies of all the individual steps leading to the conversion α.

$$E_\alpha = -R\left(\frac{\delta\left(\frac{d\alpha}{dt}\right)}{\delta T^{-1}}\right) \tag{4}$$

2.3 Isoconversional Methods for Apparent activation energy

If a sample of mass m_0 undergoes thermal decomposition under a prescribed heating program, T, then the rate of decomposition is a function of its transformation/conversion with temperature/time (α), and pressure (P) during the process. The rate of decomposition is given as

$$\frac{d\alpha}{dt} = k(T) f(\alpha) h(P) \ni \alpha = \frac{m_0 - m_t}{m_0 - m_f}\ ; T = T_0 + \beta t \tag{5}$$

Here, m_0, m_f, and m_t are the initial mass, final mass, and mass, respectively at a given time (t) as the sample decomposes. T_0 and β are the starting temperature and heating rate, respectively. α range from 0 (at start of reaction) to 1(completion of reaction) and k(T) is the rate constant that is assumed to follow the Arrhenius and vant Hoff temperature dependence of rate constants (eqn 6). Furthermore, the temperature of the heating program (Reference temperature), T_r, is assumed to be approximately equal to the sample temperature, T_s, measured by the TGA/DSC (eqn 7).

$$k = A \exp\left(-\frac{E_\alpha}{RT}\right) \tag{6}$$

$$T - T_s \approx 0 \tag{7}$$

Here, A is the apparent pre-exponential factor, R is the gas constant, T is the temperature and E_α is the apparent activation energy at α. The pressure dependence h(P) is considered negligible since the experiments are performed under a pure kinetic regime with negligible thermal gradients. By substitution, and taking natural log on both sides, we can rewrite eqn 5 as eqn 8.

$$\ln\frac{d\alpha}{dt} = \ln(f(\alpha)A) + -\frac{E}{RT} \tag{8}$$

Under the isoconversional assumption of unchanging reaction models at incremental increase in heating rates, we can say that for a heating rate (i)

$$\ln\frac{d\alpha}{dt} = \ln(f(\alpha)A) + -\frac{E_\alpha}{RT_{\alpha,i}} \tag{9}$$

The slope of ln(dα/dt) vs ($1/T_{\alpha,i}$) for different heating rates (e.g. i = 5, 7, 10, 12, 15, 20K/min) results in the apparent activation energy at α. This is Friedman's differential isoconversional method [8]. The advantage of this method is that one can directly substitute $T_{\alpha,i}$ as the sample temperature that is measured by the instrument thereby incorporating the effects of sample self-heating and exothermic transformations. However, since TGA only measures α, we must mathematically and arrive at dα/dt which can create noise and associated errors. Eqn 5 can be rewritten as eqn 10 and integrated (eqn 11 and 12)

$$\frac{d\alpha}{f(\alpha)} = A \exp\left(-\frac{E}{RT}\right) dt \tag{10}$$

$$\int_0^{\alpha} \frac{d\alpha}{f(\alpha)} = A \int_0^{t} \exp\left(-\frac{E}{RT}\right) dt \tag{11}$$

$$g(\alpha) = \frac{A}{\beta} \int_{T_0}^{T} \exp\left(-\frac{E}{RT}\right) dT \because T = T_0 + \beta t \tag{12}$$

where g(α) is the integral form of the reaction model. This can be further written as

$$g(\alpha) = \frac{A}{\beta} \left(\int_0^{T} \exp\left(-\frac{E}{RT}\right) dT - \int_0^{T_0} \exp\left(-\frac{E}{RT}\right) dT \right) \tag{13}$$

$$= \frac{A}{\beta} \left(S(T) - S(T_0) \right) \tag{14}$$

If we consider the process rate between 0 and starting temperature, T_0, to be negligible, then this eqn 13 can be rewritten as eqn 15 where I(E, T) is the temperature integral. Since I(E,T) has no analytical solution [9], integral isoconversional methods pivots around linear approximations for I(E,T).

$$g(\alpha) = \frac{A}{\beta} \int_0^{T} \exp\left(-\frac{E}{RT}\right) dT = \frac{A}{\beta} I(E,T) \tag{15}$$

Consider x=E/RT, then eqn 15 can be rewritten in terms of x between limits ∞ and x (eqn 16). And natural logarithm is taken on both sides (eqn 17 and 18).

$$g(\alpha) = \frac{AE}{\beta R} \int_x^{\infty} \exp \frac{(-x)}{x^2} dx \tag{16}$$

$$\ln g(\alpha) = ln \frac{AE}{\beta R} + \ln P(x) \qquad (17)$$

$$\ln \beta = \ln \frac{AE}{Rg(\alpha)} + \ln P(x) \tag{18}$$

Where P(x) = $\int_x^{\infty} \exp \frac{(-x)}{x^2} dx$. Based on the approximations for P(x), we arrive from eqn 18 to eqn 19, which is the general form of KAS and Starink method [10].

$$\ln \frac{\beta}{T^m} = Constant - C \frac{E}{R} \times \frac{1}{T} \tag{19}$$

Where m = 2 and c = 1 for KAS method; m=1.92 and c=1.0008 for Starink method. Evaluating the slope of ln β/T^m vs 1/T results in apparent activation energy at α. These methods do not have the drawback of signal noise, if any, compared to Friedman's technique. However, limitations are a) reference temperature is used here instead of sample temperature. This can result in errors if $T-T_s \gg 0$

is true at some α, b) these methods utilize some form of linear approximation of the temperature integral which in turn leads to the estimation of E_α that varies with the chosen approximations. These limitations can be overcome by the non-linear isoconversional (NLN) method [11]. Consider eqn 11 that can be rewritten in a time-dependent form for a particular α

$$g(\alpha) = A \int_0^{t_\alpha} \exp\left(-\frac{E_\alpha}{RT(t)}\right) dt \tag{20}$$

This reveals another critical limitation of the integral isoconversional method. Integration is done over the limits with the assumption that E_α is fairly constant (assumption of sequential reaction model). However, for complex reactions (with concurrent reactions), E_α can change with conversions leading to systematic errors. Hence, eqn 20 can be modified as an integral (eqn 21) between two conversion steps separated by Δα – a piecewise continuous integration [12].

$$g(\alpha) = A \int_{t_{\alpha-\Delta\alpha}}^{t_\alpha} \exp\left(-\frac{E_\alpha}{RT(t)}\right) dt = A\,J\big(E, T(t)\big) \tag{21}$$

Under the assumption of the isoconversional principle, g(α) would be invariant with heating program. Hence, eqn 21 can be expressed as an equality of n heating rates (eqn 22). This would be satisfied under the minimization condition (eqn 24)

$$A\,J\left(E_{alpha}, T_{\alpha,1}(t)\right) = A\,J\left(E_{alpha}, T_{\alpha,2}(t)\right) = \cdots = A\,J\left(E_{alpha}, T_{\alpha,n}(t)\right) \tag{22}$$

$$\sum_{i\neq j}^{n}\sum_{j}^{n}\frac{J\left(E_\alpha, T_{\alpha,i}(t)\right)}{J\left(E_\alpha, T_{\alpha,j}(t)\right)} = n(n-1) \tag{23}$$

$$Minimize\ (E_\alpha) = \sum_{i\neq j}^{n}\sum_{j}^{n}\frac{J\left(E_\alpha, T_{\alpha,i}(t)\right)}{J\left(E_\alpha, T_{\alpha,j}(t)\right)} \tag{24}$$

For each α, eqn 24 can be numerically solved to calculate the corresponding apparent activation energy. The eqn 24 is the NLN method and is time-dependent i.e., sample temperatures can be substituted while not possessing the concern of noise as in the differential method and not having any approximations as KAS and linear integral isoconversional methods. More detailed reviews and explanations of these isoconversional methods are available in other studies [13,14].

2.4 Preexponential factor

The preexponential factor (A) represents the probability of the existence of a transition state in thermal equilibrium with surroundings and possesses sufficient energy E_a to proceed along the reaction coordinate to products [15]. Preexponential/frequency factor (A) cannot be calculated without knowledge of the reaction model. Utilization of Master Plots methods and Invariant Kinetic Parameters methods for A can result in errors when there are considerable variations in E_a with respect to α [16]. However, A can be calculated based on pseudo kinetic compensation effect (pKCE) as a linear eqn 25

$$\ln A = a + bE_a \qquad (25)$$

Where a and b are the intercept and the slope. This implies that any changes in E_a due to slight variations in reaction conditions would not alter the reaction rate as it is compensated/offset by an increase in ln A. However, it can be a mathematical artifact that arises from the reciprocal relation between A and exp(-E/RT). True KCE is a rate behavior for a set of reactions sharing a common feature (e.g., same reactant heated under different heating rates) where the Arrhenius parameters show a compensatory (linear) relationship as shown by eqn 26. Such a "true" compensation relation exists when these set of reactions possess a temperature, T_θ (isokinetic temperature), where they exhibit the same reaction rate (k_θ). This means the Arrhenius plots of these reactions intersect at ln k_θ and T_θ – isokinetic point. Thus, eqn 25 can be rewritten as eqn 26.

$$\ln A = \frac{E_a}{RT_\theta} + lnk_\theta \qquad (26)$$

A linear relation between ln A and E_a or enthalpy and entropy changes [17] that arise from the fitting of different reaction models to an isothermal run does not necessarily lead to an isokinetic point. This is termed the false or pseudo kinetic compensation effect [18]. The physical interpretation of KCE is debatable [1] and some consider it only empirical [19]. Its detailed discussion is beyond the scope of this article and is available in the cited literature of this section.

2.5 Negative Apparent Activation Energy

Biomass pyrolysis may include a set of complex reactions that are catalytic, parallel, and reversible or non-reversible. Occurrence of $-E_\alpha$ after MPT (during decelerating mass loss stage) with associated

exothermicity have been noted in other studies [20,21] and some have attributed it to lignin [22,23] content in biomass.

$$k = A \exp\left(-\frac{E_D}{k_b T_\alpha}\right) \exp\left(-\frac{\delta F}{k_b T_\alpha}\right) \quad (27)$$

Several reaction mechanisms like nucleation, thermal annealing, and low-temperature oxidation can lead to -E_α. In nucleation reactions, the reaction rate may be expressed as eqn 27 where k, A, E_D, δF, and T_α are reaction rate, preexponential factor, activation energy for diffusion across phase boundary, max free energy for nucleus formation, and temperature at a particular conversion α respectively [24]. And k_b is the Boltzmann constant. From this eqn 27, we can see that at any α the slope of the Arrhenius plot will be - (E_D + δF). This means at those temperatures where δF is very high with a limited transport process would result in reaction deceleration or a negative apparent activation energy. Negative E_α are also encountered during low-temperature oxidation reactions [25,26], and exothermic adsorption processes where rate improves with lowering of temperature. Shifts from pure chemical regime to diffusion-control can result in the sudden transition of E_α to negative values[27]. Thermal annealing of biochar is the micro-textural changes of carbon matrix in non-graphitizing solid residue. It takes place after the primary devolatilization of biomass as the temperature and residence time are increased. These include the development and lateral growth of aromatic structures and their stacking/cross-linking. There is no strict starting point for thermal annealing. It is a wide zone that can begin during the active pyrolysis [28]. It involves parallel reactions that depend on temperature, ash content (their catalytic/inhibiting nature), and mass transfer by diffusion (during gasification stage). However, thermal annealing becomes more prevalent at higher temperatures. And its effect (i.e. decrease in reactivity of biochar) is more present in char-O_2 reaction as opposed to gasification [29]. Thus, annealed biochar from SS, BP, and AD does not necessarily show any reduced reactivity at gasification temperatures, which is also in agreement with the observations. And, the change of reactivity from non-annealed to annealed char as a sole reason for the transition of E_α from positive to negative is more likely at temperatures above 750℃ [30].

2.6 Thermodynamic Properties

With the evaluated values of E_α and A, the thermodynamic properties – change in enthalpy (ΔH_α), change in entropy (ΔS_α) and change in Gibbs free energy (ΔG_α) – at each α can be calculated based on the relationship between Eyring-Polanyi and Arrhenius equations (eqn 27-29).

$$\Delta H_\alpha = E_\alpha - RT_\alpha \quad (28)$$

$$\Delta G = E_\alpha - RT_m \ln\left(\frac{K_B T_m}{hA}\right) \quad (29)$$

$$\Delta S = \frac{\Delta H - \Delta G}{T_m} \quad (30)$$

Here R, K_B, and h are gas constant, Boltzmann constant and Planck constant respectively. T_m is peak temperature in DTG

List of Abbreviations

AD – Anaerobic digestate

A – Preexponential factor corresponding to E \alpha

BP – Banana peduncles

D-DSC – Derivative differential scanning calorimetry

DSC – Differential scanning calorimetry

DTG – Differential thermogravimetry

E_α - Apparent activation energy at conversion α

FT-IR – Fourier transform infrared spectroscopy

HeSTR – Heterogenous secondary tar reactions

HHV – Higher heating value

HoP – Heat of pyrolysis

HoSTR – Homogenous secondary tar reactions

HTT – Highest treatment temperature

KCE – Kinetic compensation effect

MPT – Maximum peak temperature

MWB – Mineral- and ash-rich waste biomass

NC – Non condensable gases

pKCE – pseudo kinetic compensation effect

SS – Sewage sludge

TGA – Thermogravimetric analyzer

α – conversion

References

1. White, J.E.; Catallo, W.J.; Legendre, B.L. Biomass pyrolysis kinetics: A comparative critical review with relevant agricultural residue case studies. *Journal of Analytical and Applied Pyrolysis* **2011**, *91*, 1-33, doi:10.1016/j.jaap.2011.01.004.
2. Vyazovkin, S.; Burnham, A.K.; Criado, J.M.; Pérez-Maqueda, L.A.; Popescu, C.; Sbirrazzuoli, N. ICTAC Kinetics Committee recommendations for performing kinetic computations on thermal analysis data. *Thermochimica Acta* **2011**, *520*, 1-19, doi:10.1016/j.tca.2011.03.034.
3. Mallick, D.; Poddar, M.K.; Mahanta, P.; Moholkar, V.S. Discernment of synergism in pyrolysis of biomass blends using thermogravimetric analysis. *Bioresour Technol* **2018**, *261*, 294-305, doi:10.1016/j.biortech.2018.04.011.
4. Diblasi, C. Modeling chemical and physical processes of wood and biomass pyrolysis. *Progress in Energy and Combustion Science* **2008**, *34*, 47-90, doi:10.1016/j.pecs.2006.12.001.
5. Mafu, L.D.; Neomagus, H.; Everson, R.C.; Strydom, C.A.; Carrier, M.; Okolo, G.N.; Bunt, J.R. Chemical and structural characterization of char development during lignocellulosic biomass pyrolysis. *Bioresour Technol* **2017**, *243*, 941-948, doi:10.1016/j.biortech.2017.07.017.
6. Wang, S.; Liu, Q.; Luo, Z.; Wen, L.; Cen, K. Mechanism study on cellulose pyrolysis using thermogravimetric analysis coupled with infrared spectroscopy. *Frontiers of Energy and Power Engineering in China* **2007**, *1*, 413-419, doi:10.1007/s11708-007-0060-8.
7. Vyazovkin, S. A time to search: finding the meaning of variable activation energy. *Phys Chem Chem Phys* **2016**, *18*, 18643-18656, doi:10.1039/c6cp02491b.
8. Friedman, H.L. Kinetics of thermal degradation of char-forming plastics from thermogravimetry. Application to a phenolic plastic. *Journal of Polymer Science Part C: Polymer Symposia* **2007**, *6*, 183-195, doi:10.1002/polc.5070060121.
9. Órfão, J.J.M. Review and evaluation of the approximations to the temperature integral. *AIChE Journal* **2007**, *53*, 2905-2915, doi:10.1002/aic.11296.
10. Starink, M.J. The determination of activation energy from linear heating rate experiments: a comparison of the accuracy of isoconversion methods. *Thermochimica Acta* **2003**, *404*, 163-176, doi:10.1016/s0040-6031(03)00144-8.
11. Vyazovkin, S. Modification of the integral isoconversional method to account for variation in the activation energy. *Journal of Computational Chemistry* **2000**, *22*, 178-183, doi:10.1002/1096-987X(20010130)22:2%3C178::AID-JCC5%3E3.0.CO;2-%23.
12. Burnham, A.K.; Dinh, L.N. A comparison of isoconversional and model-fitting approaches to kinetic parameter estimation and application predictions. *Journal of Thermal Analysis and Calorimetry* **2007**, *89*, 479-490, doi:10.1007/s10973-006-8486-1.
13. Vyazovkin, S. *Isoconversional Kinetics of Thermally Stimulated Processes*, 1 ed.; Springer, Cham: Switzerland, 2015; p. 239.
14. Cai, J.; Xu, D.; Dong, Z.; Yu, X.; Yang, Y.; Banks, S.W.; Bridgwater, A.V. Processing thermogravimetric analysis data for isoconversional kinetic analysis of lignocellulosic biomass pyrolysis: Case study of corn stalk. *Renewable and Sustainable Energy Reviews* **2018**, *82*, 2705-2715, doi:10.1016/j.rser.2017.09.113.
15. Sapunov, V.N.; Saveljev, E.A.; Voronov, M.S.; Valtiner, M.; Linert, W. The Basic Theorem of Temperature-Dependent Processes. *Thermo* **2021**, *1*, 45-60, doi:10.3390/thermo1010004.
16. Sbirrazzuoli, N. Determination of pre-exponential factors and of the mathematical functions f(α) or G(α) that describe the reaction mechanism in a model-free way. *Thermochimica Acta* **2013**, *564*, 59-69, doi:10.1016/j.tca.2013.04.015.
17. Liu, L.; Guo, Q.X. Isokinetic relationship, isoequilibrium relationship, and enthalpy-entropy compensation. *Chem Rev* **2001**, *101*, 673-695, doi:10.1021/cr990416z.
18. Koga, N. A review of the mutual dependence of Arrhenius parameters evaluated by the thermoanalytical study of solid-state reactions: The kinetic compensation effect. *Thermochimica Acta* **1994**, *244*, 1-20, doi:10.1016/0040-6031(94)80202-5.
19. L'vov, B.V.; Galwey, A.K. Interpretation of the kinetic compensation effect in heterogeneous reactions: thermochemical approach. *International Reviews in Physical Chemistry* **2013**, *32*, 515-557, doi:10.1080/0144235x.2013.802109.
20. Chen, X.; Liu, L.; Zhang, L.; Zhao, Y.; Zhang, Z.; Xie, X.; Qiu, P.; Chen, G.; Pei, J. Thermogravimetric analysis and kinetics of the co-pyrolysis of coal blends with corn stalks. *Thermochimica Acta* **2018**, *659*, 59-65, doi:10.1016/j.tca.2017.11.005.

21. Boehrk, H.; Stokes, J.L. Kinetic Parameters and Thermal Properties of a Cork-Based Material. In Proceedings of the 20th AIAA International Space Planes and Hypersonic Systems and Technologies Conference, 2015.
22. Parthasarathy, P.; Narayanan, K.S.; Arockiam, L. Study on kinetic parameters of different biomass samples using thermo-gravimetric analysis. *Biomass and Bioenergy* **2013**, *58*, 58-66, doi:10.1016/j.biombioe.2013.08.004.
23. Celebi, M.C.; Karatepe, N. An Investigation of Thermal Decomposition Behavior of Hazelnut Shells. *International Journal of Green Energy* **2014**, *12*, 93-97, doi:10.1080/15435075.2014.893876.
24. Vyazovkin, S.; Sbirrazzuoli, N. Isoconversional Analysis of Calorimetric Data on Nonisothermal Crystallization of a Polymer Melt. *The Journal of Physical Chemistry B* **2002**, *107*, 882-888, doi:10.1021/jp026592k.
25. R.G. Compton, G.H. *Low-Temperature Combustion and Autoignition*; Elsevier: England, 1997; Volume 35.
26. McCarty, J.G. Kinetics of PdO combustion catalysis. *Catalysis Today* **1995**, *26*, 283-293, doi:10.1016/0920-5861(95)00150-7.
27. Veser, G.; Wright, A.; Caretta, R. On the oxidation–reduction kinetics of palladium. *Catalysis Letters* **1999**, *58*, 199-206, doi:https://doi.org/10.1023/A:1019050522282.
28. Senneca, O.; Ontyd, C.; Cerciello, F.; Schiemann, M.; Scherer, V. Extension of the Thermal Annealing Concepts Developed for Coal Combustion to Conversion of Lignocellulosic Biomass. *Energy & Fuels* **2020**, *34*, 3661-3670, doi:10.1021/acs.energyfuels.9b03172.
29. Senneca, O.; Scala, F.; Chirone, R.; Salatino, P. Relevance of structure, fragmentation and reactivity of coal to combustion and oxy-combustion. *Fuel* **2017**, *201*, 65-80, doi:10.1016/j.fuel.2016.11.034.
30. Senneca, O.; Salatino, P. A semi-detailed kinetic model of char combustion with consideration of thermal annealing. *Proceedings of the Combustion Institute* **2011**, *33*, 1763-1770, doi:10.1016/j.proci.2010.08.011.
